# Combining Chemical Profiling and Network Analysis to Investigate the Pharmacology of Complex Prescriptions in Traditional Chinese Medicine

**DOI:** 10.1038/srep40529

**Published:** 2017-01-13

**Authors:** Tongchuan Suo, Jinping Liu, Xi Chen, Hua Yu, Tenglong Wang, Congcong Li, Yuefei Wang, Chunhua Wang, Zheng Li

**Affiliations:** 1 College of Pharmaceutical Engineering of Traditional Chinese Medicine, Tianjin University of Traditional Chinese Medicine, Tianjin 300193, PR China; 2 Tianjin Key Laboratory of Modern Chinese Medicine, Tianjin University of Traditional Chinese Medicine, Tianjin 300193, PR China

## Abstract

We present a paradigm, combining chemical profiling, absorbed components detection in plasma and network analysis, for investigating the pharmacology of combination drugs and complex formulae. On the one hand, the composition of the formula is investigated comprehensively via mass spectrometry analysis, followed by pharmacological studies of the fractions as well as the plasma concentration testing for the ingredients. On the other hand, both the candidate target proteins and the effective ingredients of the formula are predicted via analyzing the corresponding networks. The most probable active compounds can then be identified by combining the experimental results with the network analysis. In order to illustrate the performance of the paradigm, we apply it to the Danggui-Jianzhong formula (DJF) from traditional Chinese medicine (TCM) and predict 4 probably active ingredients, 3 of which are verified experimentally to display anti-platelet activity, i.e., (Z)-Ligustilide, Licochalcone A and Pentagalloylglucose. Moreover, the 3-compound formulae composed of these 3 chemicals show better anti-platelet activity than DJF. In addition, the paradigm predicts the association between these 3 compounds and COX-1, and our experimental validation further shows that such association comes from the inhibitory effects of the compounds on the activity of COX-1.

Prescriptions in traditional Chinese medicine (TCM) are well known by their adoption of “multi-chemical components” to take “multi-pharmacological effects” on “multi-action targets”^1^. However, the complicated chemical composition also brings great difficulties to the pharmacological investigations of TCM prescriptions. Network pharmacology, which was first proposed by Hopkins^2^, offers an ideal paradigm to deal with multi-target combination drugs and has recently been successfully adopted to investigate the formulae in TCM^3^,^4^,^5^,^6^,^7^. The core of the scheme is the construction and analysis of the pharmacological network, which is normally composed of the nodes of active ingredients, the nodes of candidate protein targets, the nodes of intermediate proteins transferring protein-protein interactions (PPI) and the connections (i.e., edges) between them. While the PPI can always be collected from online databases, it is essential to have the chemical composition of the prescription and the candidate protein targets *a priori* in order to build the network.

In practice, the chemical ingredients of herbs and other TCM medicinal materials may be found from several databases. However, it is not uncommon that the composition of a TCM prescription differs dramatically from the simple summation of the ingredients of each medicinal component. For example, in the work of Yang *et al*. on Xiao-Ke-An, 40 chemicals were found from relevant databases while only 20 compounds were identified experimentally^5^. Moreover, the compounds identified *in vitro* may not be able to enter the plasma, and thus could not really explain the mechanisms of these TCM formulae. Hence, it is necessary to use chemical profiling to obtain the reliable chemical constitution to construct the network and use absorbed components analysis to validate the bio-active constituents. On the other hand, the candidate targets and other relevant proteins are always collected from databases or by text mining of literature. Although this ensures the relevance of the candidate protein targets, the essentiality of each protein is often poorly assessed, especially when the physiological disorder under investigation is dominant by cascade reactions in which the topological attributes (e.g., degree, closeness, etc.) of each node are not quite related to its importance^8^. Another concern lies in the estimation of the pharmaceutical effectiveness of each chemical ingredient through the analysis of the pharmacological network. In this respect, it is common to require an effective ingredient have direct interaction with the disease-related protein targets, which could lead to neglect of compounds with indirect but significant effectiveness. In order to see it, we illustrate 2 possible interaction modes between ingredients and targets in Fig. 1. Now suppose we are trying to assess the effectiveness of compounds C1 and C2 on the disease-related target T_*x*_. As can be seen from Fig. 1(a), C1 has interaction with four proteins, only one of which is disease-related. Hence for a given dosage, the effectiveness of C1 on the disease is roughly reduced to 1/4. On the other hand, C2 does not interact with T_*x*_ but has specific interaction with T4, the manipulator of T_*x*_. Because C2 interacts with only three proteins, its effectiveness on T4 is about 1/3 for a given dosage, which can be completely transferred to T_*x*_. As a result, it can be expected that C2 is more effective than C1, although it does not affect T_*x*_ directly. Such kind of indirect but essential effectiveness has been receiving attention in the community. For example, in the method proposed by Wang *et al*., proteins indirectly related to the disease were included in the group of effective targets, although empirical parameters are needed for further quantifying the effectiveness^7^. Indeed, if we regard the chemical ingredients as information sources and the targets as sinks, assessing the effectiveness can be mapped onto the problem of information diffusion through an interaction network^9^,^10^.

With these considerations, we develop a joint paradigm for the pharmacological studies of TCM prescriptions, integrating chemicals identification and network analysis. The overall procedure is illustrated in Fig. 2. On the one hand, experiments including fractionization, mass spectrometry, pharmacological assay and absorbed components detection are performed to the TCM prescription in order to seek for the probably active chemical ingredients. On the other hand, the candidate disease targets are collected by analyzing existing disease networks (e.g., biological pathways), which are further integrated with the prescription components and the PPI to construct the drug-target (DT) network. With this network in hand, the effectiveness of each candidate ingredient can be assessed by analyzing how much “information” that emits from the ingredient can be received by the key targets, and then the active ingredients can be predicted and finally validated experimentally. The whole procedure dramatically ease the workload to extract the active chemicals from a complicated TCM prescription (always containing hundreds of chemical compounds) and hence provide an effective paradigm to study the pharmacology of TCM.

As an implementation of the paradigm, we apply it to the Danggui-Jianzhong formula (DJF), which is primarily composed of *Angelicae Sinensis Radix* (Danggui), *Cinnanmomi Cortex* (Guixin), *Licorice* (Gancao), *Paeoniae Radix Alba* (Baishao), *Zingiber Officinale Roscoe* (Shengjiang) and *Jujubae Fructus* (Dazao). In practice, DJF works as a mixture of chemical ingredients. This prescription is extensively adopted in China for gynecological disorders related to blood issues, such as primary dysmenorrhea, with its effectiveness in blood quality promoting and pain releasing. Our focus in this article is on the anti-platelet effect of the formula, especially its effectiveness on platelet aggregation. After experimentally identifying the ingredients of DJF and finding their related proteins from online databases, an elementary-signaling-mode (ESM) analysis is used on the pathway of platelet activation from the Kyoto Encyclopedia of Genes and Genomes (KEGG)^11^,^12^ to extract the disease-relevant candidate targets^8^. Furthermore, an algorithm based on the information flow through the shortest simple paths is developed to study the activity of each candidate ingredient. The active compounds from DJF are finally obtained by combining the results of network analysis and absorbed components detection. We finally perform experiments to validate the prediction and discuss the mechanisms of the action of the ingredients.

## Results and Discussion

### Identification of the chemical composition of DJF

To investigate the chemical ingredients of DJF, we firstly fractionate the formula into 27 fractions (F2–F28) via our preparative chromatography system (Pr-HPLC). Since the the chemicals within the same fraction have similar polarity, the subsequent analysis can be performed more efficiently and comprehensively. We then analyze the ingredients of each fraction via ultra performance liquid chromatography coupled with quadrupole time-of-flight mass spectrometry (UPLC-QTOF-MS). The ion chromatograms are displayed in Fig. 3. By comparing the retention time, fragmentation pathways and MS/MS spectra with the data of reference compounds or literature, a total of 134 chemicals are finally identified, which can be divided into 6 groups, i.e., flavonoid, monoterpenoid, ligustilide, terpenoid saponins, coumarin and “others”.

While the details of analyzing the MS data are beyond the scope of this article, we would only take the compounds of flavonoid as a typical case to briefly illustrate the identification procedure. Among those flavonoids, compounds of flavones, dihydroflavones, chalcones and flavanes mainly fragment at their C rings in the positive/negative ion mode, generating CO (28 Da) and CO_2_ (44 Da) fragments via the retro-Diels-Alder (RDA) reaction. For example, in the positive ion mode, the [M + H]^+^ (*m*/*z* 257.0809) ion from Liquiritigenin (No. 59 in Fig. 3) further fragments via RDA, losing one C_8_H_8_O_8_ (4-vinylphenol) and leading to the generation of abundant ions at *m*/*z* 137.0231. It can also lose one C_6_H_6_O_2_ (resorcinol) with the generation of ions at *m*/*z* 147.0441, or lose one C*O* with the generation of ions at *m*/*z* 119.0499. Meanwhile, in the negative ion mode, the [M − H]^−^ (*m*/*z* 255.0650) ion from Liquiritigenin generates ions at *m*/*z* 119.0493 and 135.0079 via RDA. On the other hand, flavone glycosides, which can be further divided into flavone O-glycosides and flavone C-glycosides, perform relatively simple fragmentation. Specifically, flavone O-glycosides usually lose glucose (162 Da), apiose (132 Da) and rhamnose (146 Da) simultaneously or consecutively, while flavone C-glycosides dehydrates at the hydroxyls of the saccharide groups. The 134 compounds are all obtained through such analysis of the MS data and summarized in Supplementary Table S1.

### Prediction of the active ingredients

Accompanying the chemical analysis of the 27 fractions, we also explore their anti-platelet activities through the platelet aggregation experiments and find 10 effective fractions (i.e., F11, F12, F14–F17, F24–F26 and F28; see Supplementary Fig. S1). For efficiency, only the 86 compounds from these 10 effective fractions are considered when constructing the DT network, 19 of which have associated proteins reported and hence constitute the set of candidate ingredients (Table 1), along with their 450 associated proteins from the relevant databases (see the section “Methods” for more details). On the other hand, the ESM analysis is performed to the pathway of platelet activation (KEGG entry ID: hsa04611), which results in 56 potential target proteins, as listed in Supplementary Table S2. Further combining these results with the PPI extracted from the Human Protein Reference Database (HPRD)^13^ and iRefIndex^14^, we obtain the final DT network (Fig. 4) for DJF. This network is composed of 1774 nodes and 13475 edges, in which the 19 candidate ingredients affect the 56 potential targets either directly or through the intermediate PPI network.

We subsequently calculate the effectiveness scores (i.e., *I(m*) values) and the specificity scores (i.e., the maximal value of *I(m* → *n*)) for these 19 ingredients via a simple algorithm, which maps the problem onto the information flow through an interaction network, as depicted in the section “Methods”. The results are summarized in Table 1. The list is ranked in the order of *I(m*) values, and the ingredients have relatively high *I(m*) value are expected to be more effective to regulate the disorder under investigation (i.e., platelet aggregation in our case). In the mean time, the specificity scores are also important when assessing the effectiveness of a given compound, since lower specificity may lead to more side effects or weaker pharmaceutical action (since less dosage can reach the key target). In practice, the thresholds for the scores of effectiveness and specificity may be case-dependent. We regard an ingredient as an effective one if its *I(m*) is greater than 0.05 and its total specificity score is larger than 0.03. Please note when there are more than one maxima in the *I(m* → *n*) of the ingredient, e.g., formononetin, the maximal values are added up as the specificity score. As a result, 7 compounds from DJF are predicted as potentially effective for platelet aggregation, as denoted in italics in Table 1.

Among these 7 ingredients, anti-platelet effects have been reported for Pentagalloylglucose^15^, (Z)-Ligustilide^16^ and Licochalcone A^17^. On the other hand, Formononetin is recognized to accelerate wound repair^18^, and interact with thrombin (UniProt ID: P00734)^19^, an essential target for regulating platelet aggregation. There are also reports for the pharmaceutical effects of Glabrone and Glyasperin C. Specifically, Glabrone is known to have anti-influenza activity (probably by inhibiting neuraminidase)^20^ and PPAR-*γ* ligand-binding ability^21^. Glyasperin C is reported as a tyrosinase inhibitor^22^ and an estrogen antagonist^23^. However, there is little information for the anti-platelet effects of these 2 chemicals. We find no literature about Glyasperin F, although TCMSP predicts its interaction with cyclooxygenase-1 (COX-1, UniProt ID: P23219). With these pieces of information in hand, we regard 4 compounds, i.e., Pentagalloylglucose, (Z)-Ligustilide, Licochalcone A and Formononetin, as the most probable candidates contributing to the anti-platelet effect of DJF.

Accompany with the network analysis, we also investigate the plasma concentration of the ingredients in DJF with a rat model, via the UPLC-QTOF-MS experiments. Since the complicated composition of the plasma brings inherent noises to the MS data, reference chemicals are used to help the identification process. Specifically, a total of 26 reference compounds are available commercially, which are used to make the reference solution for the UPLC-QTOF-MS testing. The MS data of the blank rat plasma, the reference solution and the rat plasma after oral administration of the decoction of DJF are displayed in Fig. 5. After comparing the MS data (retention time, fragments, etc.) of the rat plasma sample with those of the reference solution, 22 chemicals are identified, as listed in Table 2. It can be seen that the 4 candidate compounds are present.

### Experimental validation

In order to validate the prediction from last subsection and the whole joint paradigm to study DJF, we perform platelet aggregation experiments to investigate the anti-platelet activities of the ingredients found in the plasma concentration testing. Two types of agonist are utilized, i.e., adenosine diphosphate (ADP) and thrombin, and the corresponding activation mechanism is briefly illustrated in Fig. 6(a). It is known that ADP induces platelet activation via P2Y_1_ and P2Y_12_, which further causes a series of events including thromboxane A_2_ (TXA_2_) synthesis that further promotes the aggregation of platelets. Thrombin, on the other hand, activates platelet through PAR1 and PAR4, which directly couples to G13- and Gq-mediated signaling (for shape change and aggregation, respectively)^24^,^25^,^26^. In the platelet aggregation experiments, all of the 22 compounds that are verified by the plasma concentration testing have been tested, including the 4 predicted active ingredients (Table 2). It is worth pointing out that 10 of these 22 compounds are from the less active fractions and hence excluded before constructing the DT network. The inclusion of them in the experiments helps to check the reliability of our pretreatment for screening the fractions *a priori*. The final results are shown in the parts b and c of Fig. 6, which displays the maximum aggregation rate (MAR) of different chemicals. Figure 6(b) presents the results with ADP as the platelet agonist and Fig. 6(c) gives the results for thrombin cases. In both figures, the reagents (i.e., Brilinta and Hirudin) display expected inhibitory effects on platelet aggregation, which verifies the reliability of our experiments. It can be seen from the figure that 3 ingredients dose-dependently suppress platelet aggregation, while other inactive chemicals are not shown for brevity. Specifically, (Z)-Ligustilide, Licochalcone A and Pentagalloylglucose have inhibitory activity on ADP-activated platelet aggregation, and Pentagalloylglucose can also suppress the aggregation induced by thrombin. Namely, the chemicals that are not predicted by our network analysis display no activity in the experiments either, while 3 of the 4 predicted and under-testing compounds are verified to be active. Formononetin is the one that is predicted in Table 1 but does not have inhibitory performance experimentally. Combining with the fact that its interaction with thrombin has been validated elsewhere^19^, our experimental result indicates that interaction (binding, etc.) with a key protein does not always lead to pharmaceutical effects.

With the 3 active ingredients in hand, we would further ask whether the combination of these 3 compounds could have similar anti-platelet activity to DJF. In order to answer this question, we firstly use UPLC with diode array detector (UPLC-DAD), combined with the external standard method, to determine the content of these chemicals in DJF, obtaining a ratio of content as 40.4:1.0:117.3 (Pentagalloylglucose:Licochalcone A:(Z)-Ligustilide, Supplementary Fig. S3). Then, a 3-compound formula with this content ratio is constructed as RF_2_, and another formula with content ratio 1:1:1 (RF_1_) is also constructed for comparison. We further perform the platelet aggregation experiments (ADP-activated) with these two new recombinant formulae and DJF, the results of which are shown in Fig. 6(d). From Supplementary Table S9, we know that the mass concentrations of RF_1_ − C_1_ and RF_2_ − C_1_ are 140 *μ*g/mL and 201.25 *μ*g/mL, respectively, which is lower than DJF 250 (250 *μ*g/mL). However, it can be seen from Fig. 6(d) that, both RF_1_ − C_1_ and RF_2_ − C_1_ display better anti-platelet activity than DJF 250. Moreover, RF_1_ − C_2_ and RF_2_ − C_2_ also have better activity than DJF 250, although their mass concentrations are as low as 70 *μ*g/mL and 100.63 *μ*g/mL, respectively. These results show that the 3-compound formula can have obvious better performance than the original DJF.

In addition to predicting the active ingredients, our network analysis can also hint the key target proteins associated with each compound, as listed in the parentheses in Table 1. It can be seen that, the key target for (Z)-Ligustilide and Licochalcone A is predicted to be cyclooxygenase-1 (COX-1), which modulates the generation of prostaglandin from arachidonic acid (AA) and finally affects the generation of TXA_2_. For Licochalcone A, such prediction is consistent with the observation of its inhibitory effect on the formation of thromboxane B_2_, the metabolite of TXA_2_^17^. Nevertheless, we do not find any reference that directly indicates the association between (Z)-Ligustilide and COX-1. There are relevant studies about the modulation of (Z)-Ligustilide on cyclooxygenase-2 (COX-2)^27^,^28^,^29^, a protein that has similar function as COX-1. However, in our experiments of normal platelets, COX-2 is expected to be little expressed. On the other hand, Pentagalloylglucose is predicted by our scheme to mainly affect thrombin, which is consistent with the fact that Pentagalloylglucose is the only active ingredient in the thrombin-activated platelet aggregation experiments. Indeed, the mixed noncompetitive inhibitory effect of Pentagalloylglucose on thrombin has been verified experimentally^30^. In the mean time, the performance of this compound in the ADP-activated platelet aggregation experiments is somewhat unexpected according to the network analysis, although we notice that its inhibitory effect on COX-2 has been uncovered experimentally^31^.

In order to validate the predictions of the key target proteins for the 3 active ingredients, we perform further experiments including real-time quantitative PCR (qPCR) and COX activity assay kit. The former is to test the effects of the 3 compounds on the mRNA expression level of COX-1 and the latter is to analyze whether the compounds affect the activity of COX-1. The results are summarized in Fig. 7. Specifically, Fig. 7(a) shows the qPCR results, which indicates that (Z)-Ligustilide, Licochalcone A and Pentagalloylglucose hardly affect the mRNA expression level of COX-1. However, it can be seen from Fig. 7(b) that these 3 compounds display obvious inhibitory effects on the activity of COX-1, comparable to that of aspirin. These results not only verify the prediction of our network analysis about the target proteins, but also indicate that the 3 active ingredients affect COX-1 by suppressing the activity rather than its mRNA expression level.

## Conclusion

In this article, we combine chemical profiling and network analysis to comprehensively investigate the pharmacology of combination drugs and complex formulae. On the one hand, the chemical ingredients are totally identified experimentally to confirm their presence in the formula. On the other hand, the potential targets are determined through the analysis of the disease pathway such that their relevance is certificated. Moreover, the effectiveness of each candidate chemical ingredient is assessed via the analysis of the DT network, and the specificity of the ingredient to any target is simultaneously scored, which is further combined with the plasma concentration testing in order to efficiently extract the active chemicals from the complicated formula. In addition, the scheme can also provide information on the key targets associated with each active ingredient, which helps to decipher the mechanism of the pharmaceutical action of the compound.

As an application, we use the scheme to study a traditional Chinese medicine DJF. We fractionate the formula into 27 fractions, which makes the chemical identification procedure more efficient and allows us to perform elementary study on the pharmaceutical activities of the fractions. Combining the experimental testing and the network analysis results, we predict 4 chemicals as the most probable active ingredients. Through the subsequent platelet aggregation experiments, 3 of them are verified to have anti-platelet activity, i.e., (Z)-Ligustilide, Licochalcone A and Pentagalloylglucose. Bearing in mind that all other compounds tested in the platelet aggregation experiments display no activity, these results show that the prediction of our scheme is indeed robust. In addition, according to the network analysis, (Z)-Ligustilide and Licochalcone A modulate ADP-activated platelet aggregation primarily through COX-1 and Pentagalloylglucose reduces the thrombin-activated platelet mainly via thrombin, which are consistent with the known experiments. We further experimentally test such predictions by investigating the effects of these 3 compounds on the mRNA expression and the activity of COX-1. The results indicate that they have little modulation on the mRNA expression level of COX-1 but present strong inhibitory effects on the activity of the protein.

## Methods

### Network construction

The chemical ingredients of DJF are identified experimentally. With their names and/or chemical structures (denoted by the simplified molecular-input line-entry system (SMILES) strings) as key words, ingredient-related proteins are collected from the Traditional Chinese Medicine Systems Pharmacology Database and Analysis Platform (TCMSP)^32^ and the Search Tool for Interactions of Chemicals (STITCH)^33^.

On the other hand, disease-related proteins are collected from the pathway of platelet activation. Specifically, we choose the nodes “ADP” and “Aggregation” as starting and ending points, respectively, and perform an ESM analysis to the pathway according to the algorithm in ref. 8. The essentiality of node *ν* is assessed by the reduction in the number of ESMs following the removal of *ν*, i.e.,


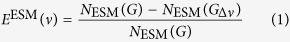


where *N*_ESM_(*G*) and *N*_ESM_(*G*_*δν*_) are the total number of ESMs from the starting point to the ending point in the original network *G* and the one after deleting *ν*, respectively. Obviously, node *ν* is essential if *E*^ESM^(*ν*) = 1, because its absence disrupts all ESMs. After the analysis, we identified 56 proteins as potential targets, as listed in Supplementary Table S2.

In addition, relevant PPI network is extracted from HPRD and iRefIndex. This along with the active ingredients, ingredient-related proteins and disease-related proteins are input into Cytoscape^34^ to build the complete DT network for DJF.

### Scoring the effectiveness of each ingredient

In order to quantitatively assess the effectiveness of each active ingredient, we now consider the question that, after emitting one unit information by one of the 19 nodes of the candidate compounds, how much effect each disease-related target can receive. We assume that the information propagates from one node to the other mainly through the simple paths connecting them in the DT network, and each node can merely and equivalently affect its neighbors, which is indeed a simple case of information flow through interaction networks^9^,^10^. Hence, if one unit of information comes to a node of degree *k*, it flows downstream through *k* − 1 branches, each of which transfers 1/*k* − 1 unit of the original information. Then, the information that target *n* receives from ingredient *m* through a simple path *i* connecting them can be evaluated as,


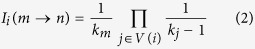


where *V(i*) = {protein nodes between *n* and *m* in path *i*} and it should be pointed out that we are considering the paths that have no other ingredient nodes than the starting point. We further take the approximation that only the *I*_*i*_(*m* → *n*) from the shortest paths are significant and thus the effectiveness of ingredient *m* on target *n* can be estimated by,


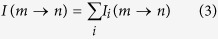


where the summation runs over all the shortest paths between *m* and *n*. The total effectiveness of ingredient *m* is given by,


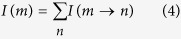


These constitute the scoring scheme for the active chemical ingredients. Specifically, *I(m*) shows the overall effectiveness of ingredient *m* on the disorder under investigation, and *I(m* → *n*) gives the specificity of ingredient *m* to target *n*.

**Figure 1:**
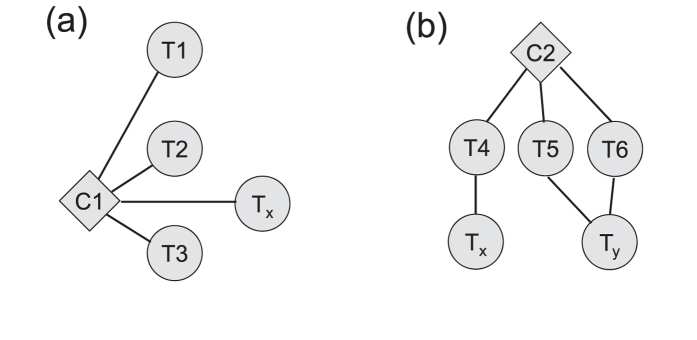
Illustration of two modes of interaction between ingredients and targets. C1 and C2 denote two chemical compounds under investigation. T1, T2, T3, T4, T5, T6, T_x_ and T_y_ denote different proteins, among which T_x_ and T_y_ are the targets that are directly related to the disease. (**a**) C1 has direct interaction with four proteins, one of which is the disease-related target T_x_; (**b**) C2 interacts with T4, T5 and T6, which further manipulates the disease-related targets T_x_ or T_y_.

**Figure 3:**
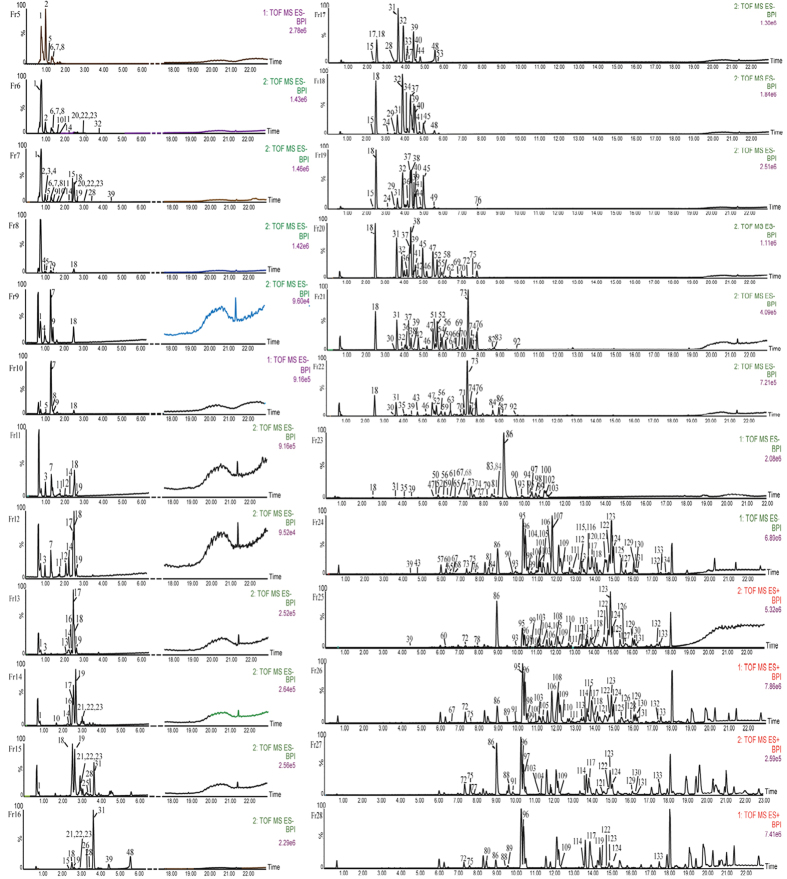
The ion chromatograms of the fractions from F5 to F28, while the signals of F2, F3 and F4 are too weak to be detected.

**Figure 4:**
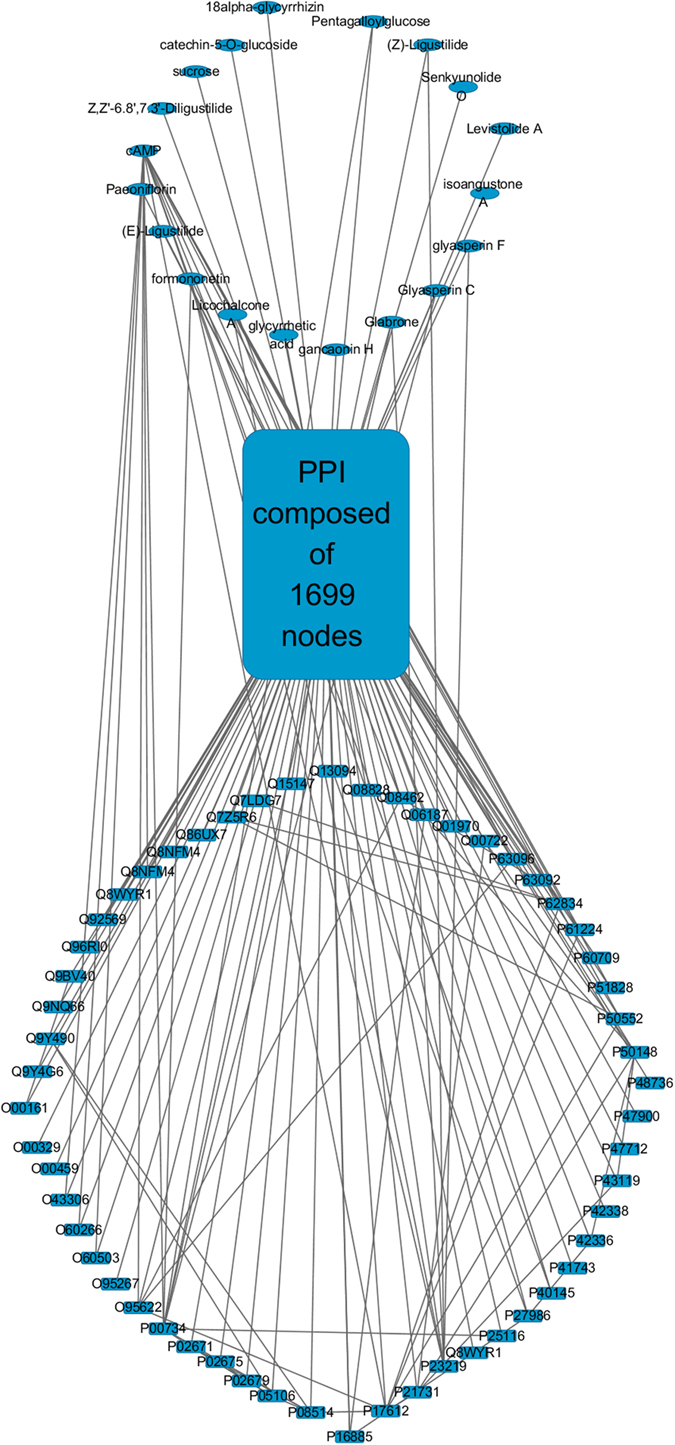
The final DT network composed of the nodes of the 19 candidate ingredients (ellipses), the 56 potential targets and the intermediate PPI.

**Figure 5:**
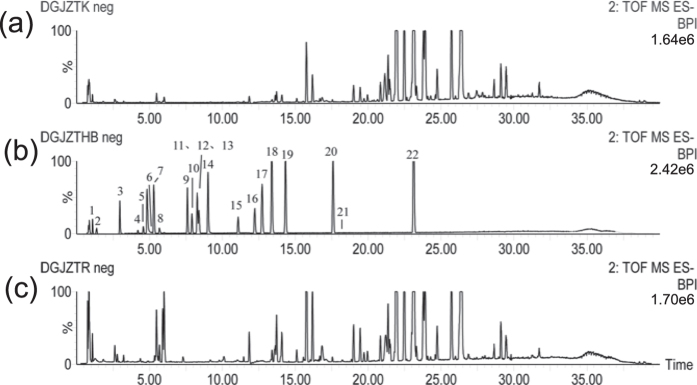
The ion chromatograms of (**a**) the blank rat plasma, (**b**) the reference solution and (**c**) the plasma sample from the rat after oral administration of DJF decoction. Negative ion mode was used during the testing.

**Figure 6:**
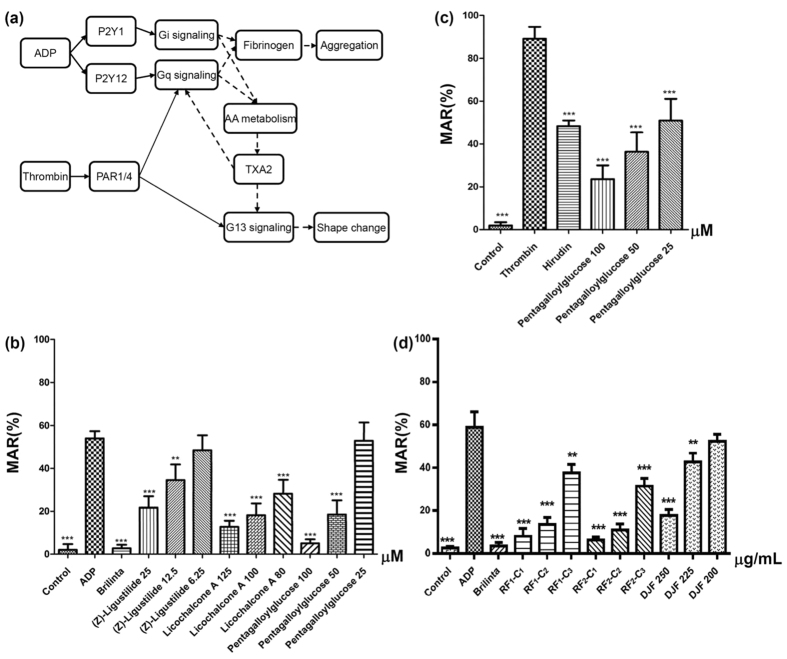
(**a**) The illustration of the platelet activation process induced by ADP and/or thrombin. The solid arrow denotes direct association, while the dashed arrow stands for indirect connection mediated by other proteins and/or compounds; (**b**) The MAR of the chemicals that have anti-platelet activity in the ADP-activated experiments; (**c**) The MAR of the chemicals that have anti-platelet activity in the thrombin-activated experiments; (**d**) The MAR of the 3-compound formulae in the ADP-activated experiments with comparison to DJF. Significant difference with respect to the corresponding agonist group: **p < 0.01; ***p < 0.001.

**Figure 7:**
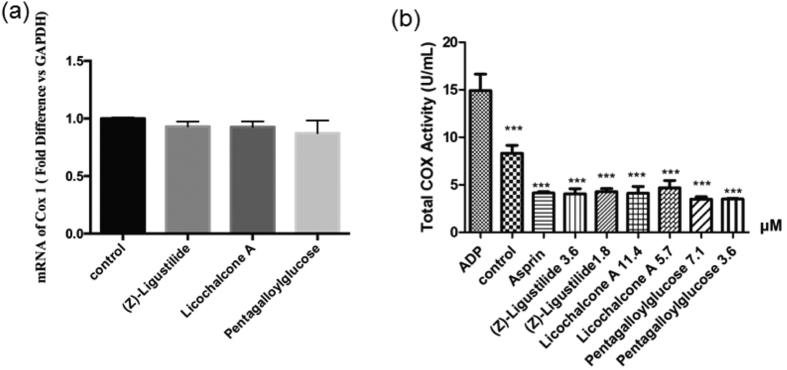
The experimental results of (**a**) the real-time quantitative PCR for detecting the mRNA level of COX-1 in the platelet treated by the active ingredients, and (**b**) the total COX activity in the platelet treated by various chemicals, with the chemical concentrations indicating in the sample names. Significant difference with respect to the corresponding agonist group: **p < 0.01; ***p < 0.001.

We can take Fig. 1(b) as an instance to illustrate the above procedure. In this example, we would like to calculate the effectiveness score of C2, i.e., *I*(C2). Because we have 2 disease-related targets T_x_ and T_y_, we need *I*(C2 → T_x_) and *I*(C2 → T_y_) to obtain *I*(C2) via Eq. 4. It can be seen that node C2 has the degree of 3 and it indirectly affects T_x_ and T_y_ through 3 mediate nodes T4, T5 and T6, the degrees of which are all 2. Since there is one shortest path connecting C2 and T_x_, i.e., C2 − T4 − T_x_, we have


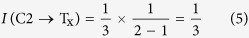


On the other hand, there are 2 shortest paths connecting C2 and T_*y*_, i.e., C2 − T5 − T_y_ and C2 − T6 − T_y_. According to Eqs 2 and 3, we have


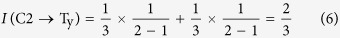


Hence, *I*(C2) is given by


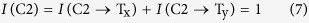


In practice, the DT network is much more complicated than Fig. 1(b), but the procedure of calculating the effectiveness score is quite similar.

### Experiments

#### Materials and chemicals

*Angelicae Sinensis Radix, Cinnanmomi Cortex, Licorice, Paeoniae Radix Alba*and *Jujubae Fructus* were purchased from Jinshaotang Herbal Medicine Co. Ltd. (Bozhou, China). *Zingiber Officinale Roscoe* was purchased from Tesco China (Tianjin, China). Pentagalloylglucose, Formononetin, Glycyrrhizin, Benzoylpaeoniflorin, Albiflorin, Isoliquiritigenin and Ononin were purchased from Yifang Technology Co. Ltd. (Tianjin, China). (Z)-Ligustilide was purchased from Jianglai-bio Co. Ltd. (Shanghai, China). Licochalcone A and Glycyrrhetic acid were purchase from Zhongxin Innova (Tianjin, China). Licorice saponin G2 was purchased from Tokiwa Phytochemical Co. Ltd. (Sakura-shi, Japan). Cyclic adenosine monophosphate (cAMP) was purchased from Harvey-bio Co. Ltd. (Beijing, China). Oxypaeoniflorin, Levistolide A, Liquiritin apioside, Glycyrrhizic acid, Ellagic acid and Neoisoliquiritin were purchased from Yuanye-bio Co. Ltd. (Shanghai, China). Sucrose was purchased from Sigma-Aldrich Co. LLC. (St. Louis, United States). Paeoniflorin, Liquiritin, Gallic acid and Citric acid were purchased from National Institutes for Food and Drug Control (Beijing, China). Isoliquiritin was purchased from Tauto Biotech Co. Ltd. (Shanghai, China). Isoliquiritin apioside was purchased from Sichuan Victory Biological Technology Co. Ltd. (Chengdu, China). Benzoyloxypaeoniflorin was purchased from Spring & Autumn Biological Engineering Co. Ltd. (Nanjing, China). Brilinta and Aspirin were purchased from Sigma-Aldrich Co. LLC. (St. Louis, MO, USA). Hirudin was purchased from GL Biochem Ltd. (Shanghai, China). TRIZOL reagent was purchased from Thermo Fisher Scientific Inc. (Carlsbad, CA, USA).

Acetonitrile and methanol, both of HPLC grade, were purchased from Sigma-Aldrich Co. LLC. (St. Louis, MO, USA). Ethanol was purchased from Real&Lead Chemical Co. Ltd. (Tianjin, China).

Sprague-Dawley rats (200~200 *g*) were purchased from the Academy of Military Science of People’s Liberation Army (Beijing, China) with production license No: SCXK-(Jun)-2012-0004. All the rats are maintained in a room equipped with an air-filtering system, and the cages and water were sterilized. The study is approved by the Research Ethics Committee of Tianjin International Joint Academy of Biomedicine. All animals are treated in accordance with the guidelines and regulations for the use and care of animals of the Center for Laboratory Animal Care, Tianjin International Joint Academy of Biomedicine.

The Quantscript RT Kit and SYBR Premix HotmasterTaq were purchased from TIANGEN Biotech (Beijing) Co., Ltd. (Beijing, China). The COX Activity Assay Kit was purchased from Cayman Chemical (Ann Arbor, MI, USA).

#### Identification of the chemical ingredients of DJF

We use UPLC-QTOF-MS system to identify the chemical ingredients of DJF and the analysis is performed through 3 main steps as follows.

Firstly, *Angelicae Sinensis Radix, Cinnanmomi Cortex, Licorice, Paeoniae Radix Alba, Zingiber Officinale Roscoe* and *Jujubae Fructus* are blended with mass ratio 4:3:2:6:3:2 and then immersed in 50% (*V*/*V*) ethanol solution with mass ratio 1:10 for 30 min. The mixture is heated under reflux for 2 hours followed by filtration. The residue is immersed in 50% (*V*/*V*) ethanol solution with mass ratio 1:8 and heated under reflux for 2 hours. After another filtration, the two filtrates are combined, distilled and dried. The obtained solid sample is mixed with 80% (*V*/*V*) methanol up to a concentration of 53 mg/mL, followed by centrifugation at 14,000 rpm for 10 min. The supernatant is further filtered with pore size of 0.24 *μ*m and the filtrate (F1) is collected for the subsequent analysis.

Secondly, F1 is fractionated via the GX-281 preparative high-performance liquid chromatography (Pr-HPLC, Gilson Inc., Middleton, WI, USA), equipped with a Agilent Prep- C_18_ column (21.2 × 250 mm, 10 *μ*m). The elution rate and injection volume are 10 mL/min and 2.0 mL, respectively. The mobile phase contains ultrapure water (A) and methanol (B), and the detailed elution condition can be found in Supplementary Table S3. A total of 27 fractions are collected with a sampling rate of 10 mL/min, which are further solidified via distillation and drying process. The sample solution of each fraction is prepared with 1 mg fraction sample well dissolved in 2 mL 50% (*V*/*V*) methanol. Each solution is then centrifuged twice at 14,000 rpm for 10 min and the supernatant is collected.

Thirdly, the sample solutions are analyzed via our UPLC-QTOF-MS platform to identify their ingredients. We use a Waters ACQUITY UPLC system (Waters Co., Milford, MA, USA) equipped with ACQUITY UPLC BEH Shield RP 18 column (100 × 2.1 mm, 1.7 *μ*m). The injection volume is 2.0 *μ*L, and the mobile phase contains 0.1% (*V*/*V*) formic acid (A) and acetonitrile (B). The detailed elution condition can be found in Supplementary Table S4. On the other hand, the operation parameters for the mass detection are: capillary voltage, −2500 V; cone voltage, 40 V; ion source temperature, 100 °C; desolvation gas (nitrogen) temperature, 400 °C; desolvation gas flow rate, 600 L/h; lower collision energy, 0 eV; upper collision energy, 15–60 eV; analysis mode, elevated-energy mass spectrometry (MS^E^).

#### Absorbed component in rat plasma assay

We immerse 80 g raw materials of DJF (16 g *Angelicae Sinensis Radix*, 12 g *Cinnanmomi Cortex*, 8 g *Licorice*, 24 g *Paeoniae Radix Alba*, 12 g *Zingiber Officinale Roscoe* and 8 g *Jujubae Fructus*) in 800 ml water for 30 min, and then boil the mixture for 2 hours. After filtration, the residue is immersed in enough water (mass ratio 1:8) and boiled for 2 hours followed by another filtration. The two filtrates are combined and distilled to a DJF solution with drug ratio 1.06 g/ml.

On the other hand, after weighing the rats, they are fasted for 12 hours with free access to water. Subsequently, we administer the DJF decoction to 6 rats, at a dose of 10 ml/kg body weight, and take blood samples from the orbital venous plexus before and after the administration (0, 5, 10, 20, 30 (min), 1, 2, 4, 6 (h)) for a total of 10 times, 0.5 ml for each time. We then put these blood samples in heparinized tubes, centrifuge them at 4 °C, 8,000 rpm for 10 min, and collect the supernatant plasma.

To analyze the DJF ingredients in the plasma, the blood samples are pretreated by mixing 100 *μ*L of each of the 10 blood samples with 300 *μ*L methanol, followed by centrifugation at 14,000 rpm for 10 min. The supernatant of each sample is dried by nitrogen at room temperature, and dissolved in 50 mL 50% (*V*/*V*) methanol. After another centrifugation at 14,000 rpm for 10 min, we collect 40 *μ*L supernatant of each sample and combined them together as the mixed plasma for the MS analysis (with the same parameters as the ingredients identification experiment). The detailed elution condition can be found in Supplementary Table S5.

#### Platelet aggregation experiment

Twenty-six of the 134 chemical ingredients of DJF are commercially available, as noted in the subsection of *Materials and chemicals*, and the corresponding solutions are prepared by separately diluting these compounds with Tris buffer solution (TBS, Supplementary Table S6) to 0.1 mM. On the other hand, platelets are taken from a male Sprague-Dawley rat (weighing 200~220 g). Whole blood obtained by arteriopuncture at the abdominal aorta of the rat is collected in a tube (about 10 ml) with 10% volume of ACD solution (Supplementary Table S7), from which the platelet-rich plasma (PRP) is isolated by 10 min centrifugation at 200 × *g* and room temperature. The PRP fraction is further processed by 10 min centrifugation at 800 × *g* and the final isolated platelets are suspended in TBS.

The grouping of the experiment samples are summarized in the Supplementary Table S8. Specifically, platelets of 100 *μ*L (about 10^7^ platelets) are added in each well of a 96-well plate, followed by adding 50 *μ*L solution of the investigated chemicals (ingredients of DJF or the reagents) or 50 *μ*L Tris buffer solution (for the negative sample), such that each well contains 150 *μ*L liquid. The plate is then incubated in a multi-mode microplate reader (FlexStation 3, Molecular Devices LLC.) for 10 min at 37 °C in order to get a well-blended platelet-chemical mixture. Subsequently, 50 *μ*L agonist solution is added to each well and the optical density (OD) value under 405 nm is measured continuously for 30 min at 37 °C. Both types of agonist solution used in our experiments, i.e., a solution of 25 *μ*M ADP and a solution of 0.1 U thrombin, contain 2.0 mM CaCl_2_.

The aggregation rate (AR) at time *t* is derived from the OD values, i.e.,


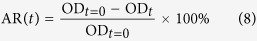


And the maximum aggregation rate (MAR) is obtained by extracting the maximum of AR(*t*) and taking the average over the wells.

In addition, we also investigate the anti-platelet activities of the 27 fractions through the above procedure, with the following distinctions:

- The concentration of each fraction solution is 225 *μ*g/mL.
- Aspirin is used as the reagent for the fractions.

#### Real-time quantitative PCR

Total RNA is extracted by using TRIZOL reagent from the platelets cultured with the active compounds (i.e., (Z)-Ligustilide, Licochalcone A and Pentagalloylglucose), and then reverse-transcribed using the Quantscript RT Kit. mRNA expression is quantified using SYBR Premix HotmasterTaq, and the gene expression of ribosomal protein large P0 is used as an internal control. Primer concentrations are 10 *μ*mol/L. The primer sequences used are as follows: GAPDH forward: 5′-GATTTGGCCGTATCGGAC-3′, reverse: 5′-GAAGACGCCAGTAGACTC-3′; Cox-1 forward: 5′-TGCATGTGGCTGTGGATGTCATCAA-3′, reverse: 5′-CACTAAGACAGACCCGTCATCTCCA-3′.

#### COX Activity Assay

We firstly activate the platelets by ADP *in vitro*, and then treat them with the active ingredients (i.e., (Z)-Ligustilide, Licochalcone A and Pentagalloylglucose) and the reagent aspirin. The total activity of COX-1 is analyzed with the COX Activity Assay Kit.

## Additional Information

**How to cite this article**: Suo, T. *et al*. Combining Chemical Profiling and Network Analysis to Investigate the Pharmacology of Complex Prescriptions in Traditional Chinese Medicine. *Sci. Rep.* **7**, 40529; doi: 10.1038/srep40529 (2017).

**Publisher's note:** Springer Nature remains neutral with regard to jurisdictional claims in published maps and institutional affiliations.

## Supplementary Material

Supplementary Information

## Figures and Tables

**Figure 2:**
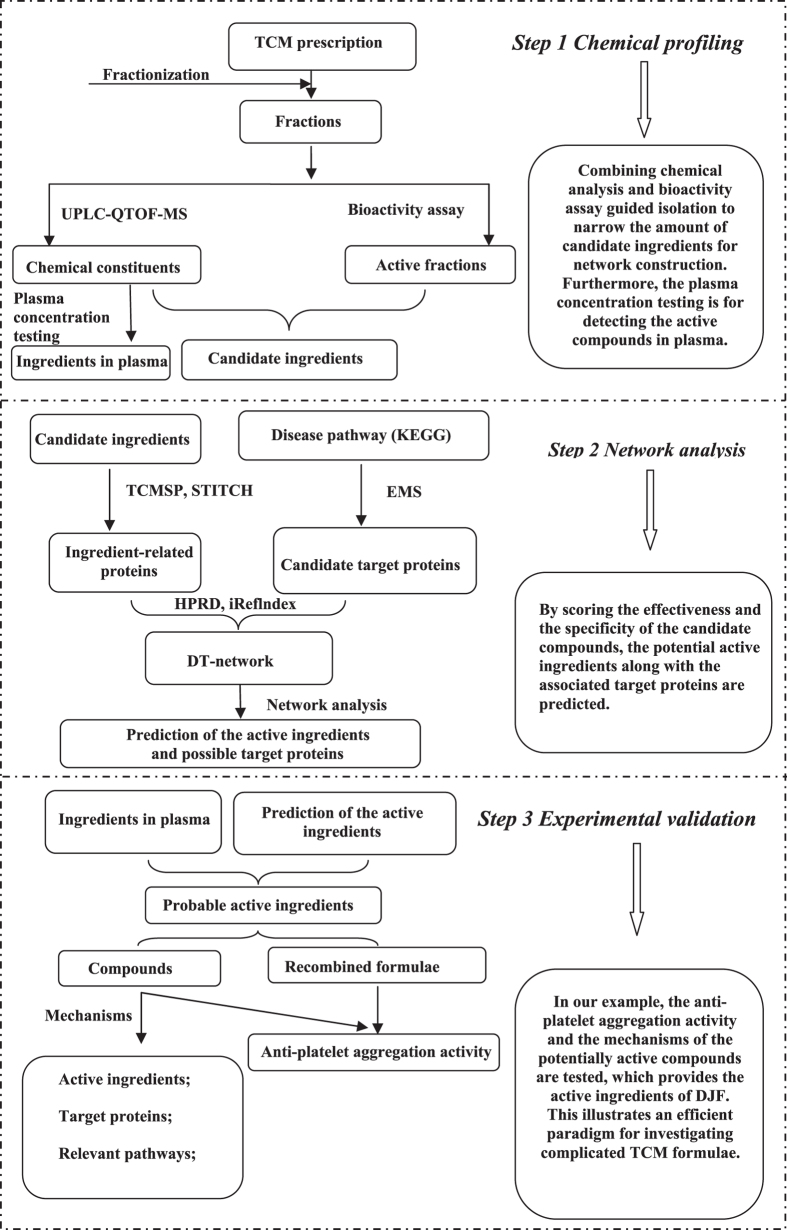
The workflow to study TCM prescriptions combining chemicals identification and network analysis.

**Table 1:** The 19 candidate ingredients and their scores of effectiveness.

Ingredient name	Max. of *I(m* → *n*)	*I(m*) value
*Pentagalloylglucose*	0.167 (P00734)^b^	0.207
*Glabrone*	0.048 (P00734 and P23219)	0.121
(*Z*)-*Ligustilide*	0.053 (P23219)	0.099
*Glyasperin C*	0.038 (P00734)	0.082
*Glyasperin F*	0.050 (P23219)	0.075
*Formononetin*	0.023 (P00734 and P23219)	0.069
cAMP^a^	0.004 (P60709)	0.067
*Licochalcone A*	0.030 (P23219)	0.058
Z,Z′-6.8′,7.3′-Diligustilide	0.011 (P17612)	0.052
Sucrose	0.021 (P17612)	0.048
Isoangustone A	0.015 (P17612)	0.047
Levistolide A	0.018 (P17612)	0.036
Gancaonin H	0.005 (P16885)	0.036
(E)-Ligustilide	0.024 (P23219)	0.035
Senkyunolide O	0.013 (P17612)	0.027
Catechin-5-O-glucoside	0.016 (P23219)	0.023
Glycyrrhetic acid	0.003 (P17612)	0.019
Paeoniflorin	—	0
18*α*-glycyrrhizin	—	0

^a^Cyclic adenosine monophosphate.

^b^The UniProt ID(s) of the target(s) corresponding to the maximum of *I(m* → *n*).

**Table 2:** Chemical information of the 22 compounds from the plasma concentration testing.

	T_R_ (min)	Formula	ES^−^ (*m*/*z*)	MS^2^	ES^+^ (*m*/*z*)	MS^2^	Identification
1	1.091	C_10_H_12_N_5_O_6_P	328.0446 (−0.3)	134.0471[ADE]^−^			cAMP
2	1.375	C_7_H_6_O_5_	169.0135 (5.3)	125.0262[M − H − CO_2_]^−^			Gallic acid
3	2.969	C_23_H_28_O_12_	495.1472 (−6.3)	137.0259[4 − OH − BZ − H]^−^			Oxypaeoniflorin
4	4.213	C_23_H_28_O_11_	525.1625[M + HCOO^−^]^−^	121.0299[BZ − H]^−^	481.1685 (−5.2)	319.1195[M + H − Glc]^+^	Albiflorin
5	4.583	C_23_H_28_O_11_	525.1614[M + HCOO^−^]^−^	449.1448[M − H − HCHO]^−^	503.1523[M + Na]	301.1110[M + H − Glc − H_2_O + H]^+^	Paeoniflorin
6	5.185	C_21_H_22_O_9_	417.1177 (−2.1)	255.0667[M − H − Glc]^−^	419.1327 (−3.6)	257.0776[M + H − Glc]^+^	Liquiritin
7	5.327	C_26_H_30_O_13_	549.1616 (1.4)	417.1204[M − H − Api]^−^	551.1766 (−5.3)	257.0788[M + H − Api − Glc]^+^	Liquiritin apioside
8	5.693	C_41_H_32_O_26_	939.1078 (−2.8)	169.016[GA − H]^−^			Pentagalloylglucose
9	7.617	C_26_H_30_O_13_	549.1638 (5.5)	417.1241[M − H − Api]^−^	551.1758 (−1.3)	257.078[M + H − Api − Glc]^+^	Isoliquritin apioside
10	7.923	C_21_H_22_O_9_	417.1193 (1.7)	255.0663[M − H − Glc]^−^	419.1311 (−7.4)	257.0795[M + H − Glc]^+^	Isoliquiritin
11	8.271	C_21_H_22_O_9_			419.1318 (−5.7)	257.0800[M + H − Glc]^+^	Neoisoliquiritin
12	8.286	C_22_H_22_O_9_	475.1277[M + HCOO^−^]^−^	267.0660[M − H − Glc]^−^	431.1333 (−2.1)	269.0769[M + H − Glc]^+^	Ononin
13	8.41	C_15_H_12_O_4_	255.0664 (2.7)	135.0101[M − H − VP]^−^		137.0207[M + H − VP]^+^	Liquiritigenin
14	9.022	C_30_H_32_O_13_	599.1772 (1.2)	137.0209[4 − OH − BZ − H]^−^			Benzoyloxypaeoniflorin
15	11.07	C_30_H_32_O_12_	629.1867[M + HCOO^−^]^−^	121.0301[BZ − H]^−^			Benzoylpaeoniflorin
16	12.229	C_15_H_12_O_4_	255.0674 (6.7)	135.0197[M − H − VP]^−^	257.0824 (3.9)		Isoliquiritigenin
17	12.734	C_16_H_12_O_4_	267.0657 (0.0)	252.0461[M − H − CH_3_]^−^	269.0813 (6.3)	237.0540[M + H − CH_3_OH]^+^	Isoliquiritigenin
18	13.382	C_42_H_62_O_17_	837.3922 (1.5)	351.0577[2GlcA − H]^−^	839.4033 (−3.8)	663.3651[M + H − GlcA]^+^	Licorice saponin G2
						487.3369[M + H − 2GlcA]^+^	
						469.3370[M + H − 2GlcA − H_2_O]^+^	
19	14.321	C_42_H_62_O_16_	821.3959 (−0.1)	351.0578[2GlcA − H]^−^	823.4133 (−4.0)	453.3361[M + H − 2GlcA − H_2_O]^+^	Glycyrrhizin
20	17.614	C_21_H_22_O_4_	337.1458 (5.3)	187.0720[M − H − 2CH_3_ − OCD]^−^			Licochalcone A
21	18.034	C_12_H_14_O_2_			191.1065 (−3.7)	173.0897[M + H − H_2_O]^+^	(Z)-ligustilide
22	23.141	C_30_H_46_O_4_	469.3343 (5.3)	355.2655[M − H − CO_2_ − ME]^−^	471.3472 (−0.4)	407.3469[M + H − HCOOH − H_2_O]^+^	Glycyrrhetic acid

Glc: Glucose, ADE: Adenine, GA: Gallic acid, BZ: Benzoic Acid, 4-OH-BZ: 4-hydroxyl-benzoic acid, VP: 4-vinylphenol, Api: apiose, GlcA: glucuronic acid, OCD: 4-oxomethylidenecyclohexa-2,5-dienone, ME: 3-methylbut-1-ene.
