# Supplementary material for: Combining Chemical Profiling and Network Analysis to Investigate the Pharmacology of Complex Prescriptions in Traditional Chinese Medicine
Source: Sci Rep. 2017 Jan 13;7:40529. doi: 10.1038/srep40529 (PMC5233960; doi:10.1038/srep40529)
Supplement: Supplementary Information [file srep40529-s1.pdf]

Combining Chemical Profiling and Network Analysis to Investigate the Pharmacology of Complex Prescriptions in Traditional Chinese Medicine

Tongchuan Suo[1,+], Jinping Liu[2,+], Xi Chen[1], Hua Yu[2], Tenglong Wang[2], Congcong Li[2], Yuefei Wang[2], Chunhua Wang[2,*], and Zheng Li[1,2,*]

[1]College of Pharmaceutical Engineering of Traditional Chinese Medicine, Tianjin University of Traditional Chinese Medicine, Tianjin 300193, PR China
[2]Tianjin Key Laboratory of Modern Chinese Medicine, Tianjin University of Traditional Chinese Medicine, Tianjin 300193, PR China
[*]Correspondence should be addressed to C.W. (pharmwch@126.com) or Z.L. (lizheng@tjutcm.edu.cn).
[+]These authors contribute equally to this work.

Supplementary information

Supplementary Table S1. Chemical information of the ingredients identified from DJF formula.

	T_R(min)	Formula	ES^-(m/z) $[M-H]^-$(ppm)	MS^2	$ES(m/z)^+$ $[M+H]^+$(ppm)	MS^2	Identification
1	0.846	$C_{12}H_{22}O_{11}$	341.1095(3.2)	179.0560$[M-H-Glc]^-$<br>161.0455$[M-H-Glc-H_2O]^-$	343.1240(0.0)	325.1138$[M+H-H_2O]^+$	Sucrose
2	1.040	$C_{10}H_{12}N_5O_6P$	328.0449(0.6)	134.0465$[ADE]^-$<br>195.0653$[DTFD]^-$	330.0596(-2.1)	136.0625$[M+H-HDTDO]^+$	cAMP
3	1.040	$C_6H_8O_7$	191.0195(1.6)	173.0086$[M-H-H_2O]^-$<br>111.0083$[M-H-CO_2-2$<br>$H_2O]^-$	193.0347(-0.5)		Citric acid
4	1.057	$C_{16}H_{24}O_{10}$	375.1293(0.5)	345.1188$[M-H-CH_2O]^-$	377.1438(-2.7)	215.0922$[M+H-Glc]^+$<br>197.0816$[M+H-Glc-H_2O]^+$	8-Diebenzoylpaeoniflorin
5	1.109	$C_{19}H_{26}O_{15}$	493.1193(0.0)	331.0663$[M-H-Glc]^-$<br>313.0559$[M-H-Glc-H_2O]^-$<br>169.0136$[GA-H]^-$	517.1171$[M+Na]^+$	315.0743$[M+H-Glc-H_2O]^+$<br>153.0182$[GA-H_2O+H]^+$	6'-o-galloylsucroses or its isomer
6	1.363	$C_7H_6O_5$	169.0135(-1.2)	125.0237$[M-H-CO_2]^-$	171.0291(-1.2)		Gallic acid
7	1.372	$C_{19}H_{26}O_{15}$	493.1200(1.4)	313.0572$[M-H-Glc-H_2O]^-$<br>169.0139$[GA-H]^-$	517.1183$[M+Na]^+$	315.0710$[M+H-Glc-H_2O]^+$<br>153.0185$[GA-H_2O+H]^+$	6'-o-galloylsucroses or its isomer
8	1.406	$C_{19}H_{26}O_{15}$	493.1194(0.2)	331.0661$[M-H- Glc]^-$<br>313.0560$[M-H-Glc-H_2O]^-$	517.1147$[M+Na]^+$	315.0714$[M+H-Glc-H_2O]^+$<br>153.0185$[GA-H_2O+H]^+$	6'-o-galloylsucroses or its isomer
9	1.458	$C_{16}H_{24}O_9$	359.1343(0.3)	197.0810$[M-H-Glc]^-$<br>179.0704$[M-H-Glc-H_2O]^-$	361.1515(4.4)	199.0969$[M+H-Glc]^+$<br>181.0863$[M+H-Glc-H_2O]^+$<br>163.0759$[M+H-Glc-2H_2O]^+$	1-o-β-D-glucopyranosyl-paeonisuf frone
10	1.658	$C_{23}H_{28}O_{14}S$	559.1129(1.3)	527.1439$[M-H-CH_2OH]^-$<br>483.0776[M-H-CH_2OH-C			Oxypaeoniflorin sulfonate

				$O_2]^-$<br>397.1323$[M-H-Glc]^-$			
11	1.806	$C_{23}H_{28}O_{14}$	527.1393(-1.5)	315.0714$[M-H-GA-Glc]^-$<br>169.0131$[GA-H]^-$			6'-o-galloyl desbenzoylpaeoniflorin isomer
12	2.104	$C_{16}H_{26}O_9$	361.1482(-4.7)			201.0472$[M+H-Glc]^+$<br>183.1027$[M+H-Glc-H_2O]^+$	6-o-β-D-glucopyranosyl-lactinolide
13	2.155	$C_{23}H_{28}O_{14}$	527.1400(-0.2)	497.1293$[M-H-HCHO]^-$<br>479.1199$[M-H-HCHO-H_2O-H]^-$<br>169.0138$[GA-H]^-$			8-o-galloyl desbenzoylpaeoniflorin
14	2.269	$C_{21}H_{24}O_{11}$	451.1232(-1.8)	289.0709$[M-H-Glc]^-$		291.0874$[M+H-Glc]^+$<br>273.0793$[M+H-Glc-H_2O]^+$	Catechin-5-o-glucoside
15	2.367	$C_{29}H_{38}O_{18}S$	705.1693(-1.1)	583.1345$[M-H-BZ]^-$<br>555.11733$[M-H-BZ-CO]^-$<br>543.1164$[M-H-Glc]^-$		501.1032$[M+H-Glc-CO_2]^+$	Isomaltopaeoniflorin sulfonate
16	2.401	$C_{23}H_{28}O_{14}$	527.1409(1.5)	497.1272$[M-H-HCHO]^-$<br>483.0770$[M-H-CO_2-H]^-$<br>313.0553$[GA+ Glc-H]^-$		333.0446$[GA+ Glc+H]^+$	6'-o-galloyl desbenzoylpaeoniflorin
17	2.504	$C_{23}H_{28}O_{12}$	495.1508(1.0)	461.1665$[M-H-2OH]^-$<br>345.1194$[M-H-BZ-CO]^-$<br>333.0984$[M-H-Glc]^-$<br>137.0240$[4-OH-BZ-H]^-$	497.1665(1.2)	335.1119$[M+H-Glc]^+$	Oxypaeoniflorin

18	2.549	$C_{23}H_{28}O_{13}S$	543.1172(0.0)	421.0798[M-H-BZ]$^-$<br>375.0751[M-H-BZ-HCOOH]$^-$<br>121.0288[BZ-H]$^-$		383.0782[M+H-Glc]$^+$<br>261.0410[M+H-Glc-BZ]$^+$	Paeoniflorin sulfonate
19	2.669	$C_{23}H_{28}O_{12}$	495.1502(-0.2)	465.0675[M-H-HCHO]$^-$<br>345.1180[M-H-BZ-CO]$^-$<br>333.1013[M-H- Glc]$^-$<br>313.0549[M-H-BZ-CH_3OH-CO]$^-$<br>137.0241[2-OH-BZ-H]$^-$		317.1034[M+H-Glc-H_2O]$^+$	Palicylpaeoniflorin
20	2.915	$C_{27}H_{24}O_{18}$	635.0890(0.9)	465.0662[M-H-GA]$^-$<br>295.0473[M-H-2GA]$^-$<br>169.0131[GA-H]$^-$		467.0325[M+H-GA]$^+$<br>449.0656[M+H-GA-H_2O]$^+$	Trigalloyglucose
21	2.967	$C_{24}H_{30}O_{13}$	525.1608(0.0)	495.1511[M-H-HCHO]$^-$<br>363.1055[M-H-Glc]$^-$<br>167.0345[HB-H]$^-$		347.1153[M+H-Glc-H_2O]$^+$	Mudanpioside E
22	3.001	$C_{21}H_{22}O_9$	417.1177(-2.2)	255.0659[M-H-Glc]$^-$<br>135.0083[M-H-VP]$^-$	419.1339(-0.7)	257.0813[M+H-Glc]$^+$<br>137.0233[M+H-VP]$^+$	Liquiritin isomer
23	3.098	$C_{32}H_{40}O_{18}$	711.2124(-1.7)	549.1597[M-H-Glc]$^-$<br>417.1163[M-H-Glc-Api-H]$^-$<br>255.0652[M-H-2Glc-Api]$^-$		419.1306[M+H-Glc-Api]$^+$<br>257.0802[M+H-2Glc-Api]$^+$	Liquiritigenin-7-o-Glucoside-4'-o-Apiosyl-o-Glucoside
24	3.167	$C_{20}H_{16}O_{13}$	463.0514(0.2)	300.9979 [M-H-Glc]$^-$	465.0673(0.9)	303.0128[M+H-Glc]$^+$	Ellagic acid-hexoside
25	3.247	$C_{29}H_{38}O_{16}$	641.2088(0.9)	519.1747[M-H-BZ]$^-$<br>489.1619[M-H-BZ-HCHO]		481.1619[M+H-Glc]$^+$<br>319.1142[M+H-2Glc]$^+$	6'-o-α-glucopyranosyl paeoniflorin

				-			
26	3.333	$C_9H_{10}O_3$	165.0549(-1.8)	121.0288[BZ-H]$^-$<br>150.0320[M-H-CH_3]$^-$<br>137.0237[M-H-CO]$^-$	167.0702(-3.6)	149.0600[M+H-H_2O]$^+$<br>121.0285[M+H-HCOOH]$^+$	Phloretic acid
27	3.378	$C_{30}H_{32}O_{15}$	631.1653(-1.6)	495.1494[M-H-MB]$^-$<br>313.0561[MG-H]$^-$<br>169.0133[GA-H]$^-$		315.0723[MG+H]$^+$<br>153.0184[GA+H-H_2O]$^+$	Galloylpaeoniflorin or its isomer
28	3.430	$C_{34}H_{28}O_{22}$	787.0996(0.3)	635.0877[M-H-GA+H_2O]$^-$<br>465.0655[M-H-2GA+H_2O]$^-$<br>449.1075[M-H-2GA]$^-$<br>313.0581[M-H-3GA+2H_2O]$^-$<br>169.0139[GA-H]$^-$		771.1032[M+H-H_2O]$^+$<br>619.0950[M+H-GA]$^+$<br>153.0184[GA+H-H_2O]$^+$	1,2,3,6-tetra-o-galloyl-β-D-glucose
29	3.527	$C_{18}H_{24}O_{11}$	415.1237(-0.7)	327.1067[M-H-2CO_2]$^-$<br>121.0287[BH-H]$^-$		267.0866[M+H-Arab]$^+$	2-o-[α- arabinopyranosyl- (1-6) -β-D-glucopyranoside]-Benzaldehyde
30	3.578	$C_{30}H_{32}O_{17}S$	695.1267(-2.2)	543.1160[M-H-GA+H_2O]$^-$<br>313.0591[GA +Glc-H_2O-H]$^-$		315.0689[GA +Glc-H_2O+H]$^+$	Galloylpaeoniflorin sulfonate
31	3.647	$C_{23}H_{28}O_{11}$	479.1553(0.0)	435.1650[M-H-CO_2]$^-$<br>357.1184[M-H-BZ]$^-$<br>327.1082[M-H-CH_2O-BZ]$^-$<br>317.1027[M-H-Glc]$^-$	481.1715(1.0)	463.1615[M+H-H_2O]$^+$<br>319.1186[M+H-Glc]$^+$<br>301.1080[M+H-Glc-H_2O]$^+$	Albiflorin

				121.0291[BZ-H]$^-$			
32	3.944	$C_{23}H_{28}O_{11}$	479.1553(0.0)	449.1448[M-H-HCHO]$^-$<br>357.1186[M-H-BZ]$^-$<br>327.1080[M-H-CH$_2$O-BZ]$^-$<br>121.0292[BZ-H]$^-$		463.1604[M+H-H$_2$O]$^+$<br>301.1075[M+H-Glc-H$_2$O+H]$^+$	Paeoniflorin
33	4.093	$C_{34}H_{28}O_{22}$	787.0984(-1.3)	617.0774[M-H-GA]$^-$<br>449.1446[M-H-2GA]$^-$<br>169.0139[GA-H]$^-$		619.0937[M+H-GA]$^+$<br>449.0680[M+H-2GA]$^+$<br>153.0181[GA+H-H$_2$O]$^+$	1,3,4,6-tetra-o-galloyl-β-D-glucose
34	4.127	$C_{21}H_{22}O_{10}$	433.1132(-0.7)	271.0613[M-H-Glc]$^-$<br>255.0657[M-H-Glc-OH]$^-$		273.0760[M+H-Glc]$^+$	5-hydroxylliquiritin
35	4.131	$C_{14}H_6O_8$	300.9989(1.7)	243.8993[M-H-CO-HCHO]$^-$<br>225.9262[M-H-H$_2$O-CO-HCHO]$^-$<br>222.8290[M-H-CO$_2$-2OH]$^-$		287.8913[M+H-CH$_3$]$^+$<br>241.8830[M+H -CO$_2$-OH]$^+$	Ellagic acid
36	4.207	$C_{27}H_{30}O_{14}$	577.1565(1.4)	517.1263[M-H-C$_2$H$_4$O$_2$]$^-$<br>487.1241[M-H-C$_3$H$_6$O$_3$]$^-$<br>457.1148[M-H-C$_4$H$_8$O$_4$]$^-$	579.1732(3.1)	561.1618[M+H-H$_2$O]$^+$<br>543.1476[M+H-2H$_2$O]$^+$<br>525.1397[M+H-3H$_2$O]$^+$	Isoviolanthin
37	4.327	$C_{26}H_{30}O_{13}$	549.1614(1.1)	417.1184[M-H-Api]$^-$<br>255.0661[M-H-Api-Glc]$^-$<br>135.0083[M-H-Api-Glc-VP]$^-$	551.1766(0.2)	257.0817[M+H-Api-Glc]$^+$<br>419.1335[M+H-Api]$^+$<br>137.0237[M+H-Api-Glc-VP]$^+$	Liquiritin apioside
38	4.407	$C_{21}H_{22}O_9$	417.1187(0.2)	255.0660[M-H-Glc]$^-$<br>135.0083[M-H-VP]$^-$	419.1335(-1.7)	257.0814[M+H-Glc]$^+$<br>137.0235[M+H-VP]$^+$	liquiritin
39	4.493	$C_{41}H_{32}O_{26}$	939.1093(-1.2)	769.0878[M-H-GA]$^-$		771.0992[M+H-GA]$^+$	Pentagalloylglucose

				617.0768[M-H-2GA+H_2O]$^-$		431.0567[M+H-3GA]$^+$	
				169.0136[GA-H]$^-$		153.0184[GA+H-H_2O]$^+$	
40	4.573	$C_{30}H_{32}O_{15}$	631.1666(0.5)	479.1559[M-H-GA+H_2O]$^-$	633.1809(-1.6)	503.1543[M+Na-GA+H_2O]$^+$	Galloylpaeoniflorin
				465.1393[M-H-BZ-CO_2]$^-$		319.1180[M+H-GA+H_2O-Glc]$^+$	
				313.0557[GA+Glc-H_2O-H]$^-$		153.0185[GA+H-H_2O]$^+$	
41	4.642	$C_{30}H_{32}O_{15}$	631.1666(0.5)	613.1561[M-H-H_2O]$^-$		615.1668[M+H-H_2O]$^+$	4'-o-galloylpaeoniflorin
				491.1195[M-H-H_2O-BZ]$^-$		493.1360[M+H-H_2O-BZ]$^+$	
				449.1448[M-H-GA-HCHO+H_2O]$^-$		315.0717[GA+Glc-H_2O+H]$^+$	
				313.0563[GA+Glc-H_2O-H]$^-$			
42	4.773	$C_{33}H_{38}O_{18}$	721.1987(1.0)	631.1658[M-H-$C_3H_6O_3$]$^-$	723.2150(1.9)	705.2054[M+H-H_2O]$^+$	Apigenin-6-C-α-L-rhamnopuranoside-8-C-[6'''-(3-hydroxy-3-methyl glutaroyl)-β-D-glucopyranoside]
				463.2186[M-H-2$C_4H_8O_4$-H_2O]$^-$		687.2048[M+H-2H_2O]$^+$	
						669.1925[M+H-3H_2O]$^+$	
						487.2126[M-HM-$C_3H_6O_3$+H]$^+$	
43	4.825	$C_{30}H_{32}O_{15}$	631.1664(0.2)	479.1554[M-H-GA+H_2O]$^-$	633.1827(1.3)	503.1547[M+Na-GA+H_2O]$^+$	6'-o-galloylalbiflorin
				449.1449[M-H-GA-HCHO+H_2O]$^-$		319.1184[M+H-GA+H_2O-Glc]$^+$	
				169.0139[GA-H]$^-$		153.0189[GA-H_2O+H]$^+$	
44	5.019	$C_{30}H_{32}O_{15}$	631.1668(0.8)	479.1561[M-H-GA+H_2O]$^-$	633.1823(0.6)	471.1298[M+H-Glc]$^+$	4-o-galloylalbiflorin

				$169.0138[GA-H]^-$		$301.1074[M+H-Glc-GA]^+$<br>$153.0187[GA-H_2O+H]^+$	
45	5.236	$C_{21}H_{22}O_{10}$	433.1133(-0.5)	$271.0603[M-H-Glc]^-$<br>$151.0029[M-H-Glc-VP]^-$		$273.0754[M+H-Glc]^+$<br>$153.0186[M+H-Glc-VP]^+$	Naringenin-7-o-β-D-glucoside
46	5.568	$C_{26}H_{30}O_{13}$	549.1616(1.5)	$417.1185[M-H-Api]^-$<br>$255.0665[M-H-Api-Glc]^-$<br>$135.0087[M-H-Api-Glc-VP]^-$	551.1766(0.2)	$419.1333[M+H-Api]^+$<br>$257.0813[M+H-Api-Glc]^+$<br>$137.0233[M+H-Api-Glc-VP]^+$	Isoliquritin apioside
47	5.602	$C_{23}H_{28}O_{11}$	479.1559(1.3)	$357.1192[M-H-BZ]^-$<br>$327.1069[M-H-CH_2O-BZ]^-$<br>$121.0292[BZ-H]^-$	481.1703(-1.5)	$319.1190[M+H-Glc]^+$<br>$301.1094[M+H-Glc-H_2O]^+$	Isopaeoniflorin
48	5.619	$C_{37}H_{36}O_{19}$	783.1761(-1.5)	$631.1647[M-H-GA+H_2O]^-$<br>$479.1552[M-H-2GA+2H_2O]^-$<br>$313.0554[GA-H_2O+Glc-H]^-$<br>$169.0133[GA-H]^-$	785.1942(1.7)	$503.1532[M+Na-2GA+2H_2O]^+$<br>$481.1733[M+H-2GA+2H_2O]^+$<br>$319.1193[M+H-2GA+2H_2O-Glc]^+$<br>$301.1072[M+H-2GA+H_2O-Glc]^+$	3',6'-di-0-galloylpaeoniflorin
49	5.655	$C_{48}H_{74}O_{21}$	985.4636(-0.8)	$954.6343[M-H-OCH_3]^-$;<br>$821.3953[M-H-Rha-H_2O]^-$	987.4797(-0.4)	$841.4497[M+H-Rha]^+$<br>$823.2448[M+H-Rha-H_2O]^+$	Yunganoside G1
50	5.682	$C_{21}H_{36}O_{11}$	463.2187(1.7)	$331.1761[M-H-Api]^-$	$487.2132[M+Na]^+$	$333.1894[M+H- Api]^+$	Zansiumaloside A
51	5.734	$C_{26}H_{30}O_{13}$	549.1608(0.0)	$417.1183[M-H-Api]^-$<br>$255.0657[M-H-Api-Glc]^-$<br>$135.0083[M-H-Api-Glc-VP]^-$		$257.0813[M+H-Api-Glc]^+$<br>$419.1342[M+H-Api]^+$<br>$137.0237[M+H-Api-Glc-VP]^+$	Licuraside

52	5.785	$C_{21}H_{22}O_9$	417.1184(-0.5)	255.0658[M-H-Glc]$^-$<br>135.0082[M-H-Glc-VP]$^-$	419.1326(-3.8)	257.0817[M+H-Glc]$^+$<br>137.0237[M+H-VP-Glc]$^+$	Isoliquritin
53	5.882	$C_{16}H_{12}O_4$	267.0665(3.0)	252.0400[M-H-CH_3]$^-$	269.0813(-0.4)	254.0460[M+H-CH_3]$^+$<br>237.0555[M+H-CH_3OH]$^+$<br>201.0464[M+H-CH_3OH-2H_2O]$^+$	Pallidiflorin
54	5.951	$C_{21}H_{22}O_9$	417.1172(-3.4)	255.0655[M-H-Glc]$^-$<br>135.0080[M-H-VP-Glc]$^-$	419.1335(-1.7)	257.0816[M+H-Glc]$^+$<br>137.0238[M+H-VP-Glc]$^+$	Neoisoliquiritin
55	5.968	$C_{35}H_{36}O_{15}$	695.1976(0.0)	549.1603[M-H-PC]$^-$		419.1347[M+H-PC-Api]$^+$	Licorice-glycoside D2 or its isomer
				531.1499[M-H-PC-H_2O]$^-$<br>417.1186[M-H-PC-Api]$^-$<br>255.0657[M-H-PC-Api-Glc]$^-$		257.0817[M+H-PC-Api-Glc]$^+$	
56	6.014	$C_{48}H_{74}O_{20}$	969.4691(-0.4)	793.4326[M-H-GlcA]$^-$		795.4532[M+H-GlcA]$^+$	3-o-[β-D-rhamnose]-24-hydroxyl-glycyrrhizic acid isomer
				497.1135[Rha+2GlcA-H]$^-$		601.4107[M-2GlcA-H_2O+H]$^+$	
57	6.031	$C_{22}H_{22}O_9$		267.0654[M-H-Glc]$^-$<br>252.0420]M-H-Glc-CH_3]$^-$	431.1331(-2.6)	269.0814[M+H-Glc]$^+$<br>254.0569[M+H-Glc-CH_3]$^+$	Ononin
58	6.065	$C_{36}H_{38}O_{16}$	725.2077(-0.7)	549.1597[M-H-PM]$^-$<br>531.1508[M-H-PM-H_2O]$^-$<br>255.0656[M-H-PM-Api-Glc]$^-$			Licorice glycoside A
59	6.214	$C_{15}H_{12}O_4$	255.0650(-2.7)	135.0080[M-H-VP]	257.0809(-1.9)	137.0238[M+H-VP]$^+$<br>147.0455[M+H-RL]$^+$<br>119.0504[M+H-RL-CO]$^+$	Liquiritigenin

60	6.300	$C_{42}H_{62}O_{18}$	853.3873(1.8)	351.0569[2GlcA-H]	855.4020(0.7)	765.4501[M+H-HCOOH-CO_2]$^+$<br>601.4045[M+H-GlcA-HCOOH-CH_3OH]$^+$<br>485.3323[M+H-2GlcA-H_2O]$^+$	22-Hydroxyl-licorice-saponin G2
61	6.380	$C_{16}H_{12}O_6$	299.0568(4.0)	255.0673[M-H-CO_2]$^-$	301.0716(1.3)	270.8882[M+H-OCH_3]$^+$<br>257.0803[M+H-CO_2]$^+$	Kaempferol 3-o-methyl ether
62	6.431	$C_{35}H_{36}O_{15}$	695.1968(-1.2)	549.1606[M-H-PC]$^-$<br>531.1500[M-H-PC-H_2O]$^-$<br>417.1193[M-H-PC-Api]$^-$<br>255.0653[M-H-PC-Api-Glc]$^-$		257.0812[M+H-PC-Api-Glc]$^+$<br>147.0442[M+H-PC-Api-Glc-RL]$^+$	Licorice-glycoside D2 or its isomer
63	6.528	$C_{21}H_{34}O_{10}$	445.2081(1.6)	313.1642[M-H-Api]$^-$<br>293.0873[Api+Glc-H]$^-$		429.1938[M+H-H_2O]$^+$	(1S,5R)-β-pinene-10-β-vicianoside
64	6.528	$C_{30}H_{32}O_{13}$	599.1766(0.2)	477.1406[M-H-BZ]$^-$<br>283.0605[BZ+ Glc-H]$^-$<br>137.0241[4-OH-BZ-H]$^-$		285.0762[BZ+ Glc+H]$^+$	Benzoyloxypaeoniflorin
65	6.606	$C_{42}H_{64}O_{16}$	823.4105(-1.3)	351.0581[2GlcA-H]$^-$	825.4235(-4.6)	649.3922[M+H-GlcA]$^+$<br>585.4132[M+H-HCOOH-H_2O]$^+$	Licorice-saponin J2
66	6.745	$C_{35}H_{36}O_{15}$	695.1981(0.7)	549.1617[M-H-PC]$^-$<br>531.1511[M-H-PC-H_2O]$^-$<br>255.0660[M-H-PC-Api-Gl		257.0822[M+H-PC-Api-Glc]$^+$	Licorice glycoside B

				c]$^-$			
67	6.713	$C_{42}H_{64}O_{16}$	823.4116(0.0)	351.0570[2GlcA-H]$^-$		649.3967[M+H-GlcA]$^+$<br>455.3535[M+H-2GlcA-H$_2$O]$^+$	Uralsaponin C
68	6.789	$C_{42}H_{62}O_{17}$	837.3875(-4.1)	351.0562 [2GlcA-H]$^-$,<br>289.0730<br>[2GlcA-H$_2$O-CO$_2$-H]$^-$	839.4051(-1.7)	663.3754[M+H-GlcA]$^+$<br>487.3426[M+H-2GlcA]$^+$<br>469.3330[M+H-2GlcA-H$_2$O]$^+$	24-Hydroxyl-glycyrrhizin
69	6.843	$C_{48}H_{72}O_{22}$	999.4428(-0.9)	837.3889[M-H-Glc]$^-$<br>351.0555[2GlcA-H]$^-$	1001.4576(-1.7)	825.4209[M+H-GlcA]$^+$<br>649.3965[M+H-2GlcA]$^+$<br>631.3855[M+H-2GlcA-H$_2$O]$^+$<br>487.3429[M+H-2GlcA-Glc]$^+$<br>469.3321[M+H-2GlcA-Glc-H<br>2O]$^+$	24-Hydroxyl-licoricesaponinA3
70	7.060	$C_{44}H_{64}O_{19}$	895.3948(-1.8)	351.0562[2GlcA-H]$^-$		545.3487[M+H-2GlcA]$^+$<br>527.3367[M+H-2GlcA-H$_2$O]$^+$	Uralsaponin F
71	7.226	$C_{42}H_{62}O_{18}$	853.3848(-1.2)	351.0561[2GlcA-H]$^-$	855.4004(-1.2)	503.3370[M+H-2GlcA]$^+$<br>485.3263[M+H-2GlcA-H$_2$O]$^+$<br>467.3147[M+H-2GlcA-2H$_2$O]<br>$^+$	22-Hydroxyl-licorice-saponin G2
72	7.340	$C_{48}H_{72}O_{21}$	983.4476(-1.2)	821.3977[M-H-Glc]$^-$<br>351.0550[2GlcA-H]$^-$	985.4650(0.6)	809.4406[M+H-GlcA]$^+$<br>615.3862[M+H-GlcA-H$_2$O]$^+$<br>453.3372[M+H-2GlcA-Glc-H$_2$<br>O]$^+$	Licorice saponin A3
73	7.391	$C_{30}H_{32}O_{12}$	583.1817(0.2)	461.1450[M-H-BZ]$^-$<br>121.0291[BZ-H]$^-$		445.1501[M+H-BZ-H$_2$O]$^+$<br>427.1391[M+H-BZ-2H$_2$O]$^+$<br>301.1074[M+H-BZ-Glc]$^+$	Benzoylpaeoniflorin

						267.0871[BZ+ Glc-H_2O+H]$^+$	
74	7.540	$C_{30}H_{32}O_{12}$	583.1812(-0.7)	121.0289[BZ-H]$^-$	585.1970(-0.3)	319.1181[M+NA-BZ-Glc-CO_2]$^+$<br>301.1076[M+H-BZ-Glc]$^+$<br>267.0871[BZ+ Glc-H_2O+H]$^+$<br>249.0764[BZ+Glc-2H_2O+H]$^+$	Isobenzoylpaeoniflorin
75	7.626	$C_{44}H_{64}O_{18}$	879.4022(0.9)	351.0559[2GlcA-H]$^-$		705.3859[M+H-GlcA]$^+$<br>511.3423[M+H-2GlcA-H_2O]$^+$<br>451.3203[M+H-2GlcA-H_2O-AceOH]$^+$	22-Acetoxylglycyrrhizin
76	7.854	$C_{42}H_{62}O_{17}$	837.3898(-1.3)	661.3580[M-H-GlcA]$^-$<br>351.0556[2GlcA-H]$^-$	839.4050(-1.8)	663.3726[M+H-GlcA]$^+$<br>469.3322[M +H-2GlcA-H_2O]$^+$	Isolicorice saponin G2
77	7.969	$C_{42}H_{62}O_{17}$	837.3915(0.7)	351.0585[M-H-2GlcA]$^-$	839.4075(1.2)	663.3738[M+H-GlcA]$^+$,<br>487.3447[M+H-2GlcA]$^+$,<br>469.3324[M+H-2GlcA-H_2O]$^+$	Yunganoside K2
78	8.003	$C_{48}H_{74}O_{20}$	969.4692(-0.3)	823.4121[M-H-Rha]$^-$<br>497.1143[2GlcA+Rha-H]$^-$		825.4094[M+H-Rha]$^+$<br>795.4512[M+H-GlcA]$^+$	3-o-[β-D-rhamnose]-24-hydroxyl-glycyrrhizic acid
79	8.224	$C_{15}H_{12}O_4$	255.0673(1.6)	135.0092[M-H-VP]$^-$<br>119.0509[VP-H]$^-$		147.0465[M+H-RL]$^+$<br>137.0249[M+H-VP]$^+$	Isoliquiritigenin
80	8.500	$C_{42}H_{62}O_{17}$	837.3906(-0.4)	819.3802[M-H-H_2O]$^-$<br>775.3896[M-H-H_2O-CO_2]$^-$<br>351.0566[2GlcA-H]$^-$	839.4058(-0.8)	663.3759[M+H-GlcA]$^+$<br>487.3425[M+H-2GlcA]$^+$<br>469.3321[M+H-2GlcA-H_2O]$^+$<br>451.3215[M+H-2GlcA-2H_2O]$^+$	Licorice saponin G2
81	8.586	$C_{16}H_{12}O_4$	266.0667(2.7)	252.0426[M-H-CH_3]$^-$	269.0826(4.5)	237.0558[M+H-CH_3OH]$^+$	Formononetin

				224.0464[M-H-CH_3-CO]$^-$<br>223.0409[M-H-CH_3-CHO]$^-$		226.0627[M+H-CH_3-CO]$^+$	
82	8.683	$C_{48}H_{72}O_{20}$	967.4517(-2.3)	351.0561[2GlcA-H]$^-$		823.4158[M+H-Rha]$^+$<br>647.3723[M+H-Rha-GlcA]$^+$<br>471.3460[M+H-Rha-2GlcA]$^+$<br>453.3364[M+H-Rha-2GlcA-H_2O]$^+$	Rhaoglycyrrhizin
83	8.718	$C_{42}H_{62}O_{17}$	837.3906(-0.4)	661.3574[M-H]-GlcA]$^-$<br>351.0569[2GlcA-H]$^-$		663.3690[M+H- GlcA]$^+$<br>487.3421[M+H-2GlcA]$^+$<br>469.3312[M+H-2GlcA-H_2O]$^+$	Macedonoside B
84	8.751	$C_{42}H_{62}O_{16}$		351.0578[2GlcA-H]$^-$	823.4081(-4.3)	647.3756[M+H-GlcA]$^+$<br>471.3464[M+H-2GlcA]$^+$<br>453.3356[M+H-2GlcA-H_2O]$^+$	Licorice saponin H2
85	8.786	$C_{44}H_{64}O_{17}$	863.4056(-1.0)	583.1813[M-H-GlcA-AceOH-CO_2]$^-$<br>351.0557[2GlcA-H]$^-$	865.4239(2.0)	689.3988[M+H-GlcA]$^+$<br>495.3459[M+H-2GlcA-H_2O]$^+$	22-Acetoxyl glycyrrhaldehyde
86	9.015	$C_{42}H_{62}O_{16}$	821.3946(-1.7)	645.3725[M-H-GlcA]$^-$<br>351.0559[2GlcA-H]$^-$	823.4142(3.2)	647.3801[M+H-GlcA]$^+$<br>453.3367[M+H-2GlcA-H_2O]$^+$<br>471.3483[M+H-2GlcA]$^+$	Glycyrrhizin
87	9.215	$C_{25}H_{32}O_9$	475.1971(0.6)	415.1762[M-H-AceOH]$^-$<br>355.1543[M-H-2AceOH]$^-$<br>295.1696[M-H-PE]$^-$	499.1912[M+Na]$^+$	417.1903[M+H-AceOH]$^+$<br>357.1695[M+H-2AceOH]$^+$	3,5-Diacetoxy-1-(4-hydroxy-3-methoxyphenyl)-7-(3,4-dihydroxy-5-methoxyphenyl)heptane
88	9.629	$C_{42}H_{64}O_{15}$	807.4144(-2.8)	631.3853[M-H-GlcA]$^-$	809.4285(-4.6)	633.3987[M+H-GlcA]$^+$,	22-dehydroxyl uralsaponinC

						457.3666[M+H-2GlcA]$^+$<br>439.3572[M+H-2GlcA-H_2O]$^+$<br>421.3664[M+H-2GlcA-2H_2O]$^+$	
89	9.661	$C_{42}H_{62}O_{16}$	821.3950(-1.2)	351.0552[2GlcA-H]$^-$	823.4147(3.7)	647.3833[M+H-GlcA]$^+$<br>471.3437[M+H-2GlcA]$^+$<br>453.3353[M+H-2GlcA-H_2O]$^+$	18α-glycyrrhizin
90	9.895	$C_{21}H_{22}O_5$	353.1382(-2.0)	338.1149[M-CH_3-H]$^-$<br>295.0597[M-H-2CH_3-CO]$^-$	355.1537(-2.3)	299.0947[M+H-BT]$^+$<br>189.0934[M-H-MVD]$^+$	Licoagrochalcone D
91	9.924	$C_{42}H_{62}O_{16}$	821.3934(-3.2)	351.0574[2GlcA-H]$^-$	823.4133(2.1)	647.3770[M+H- GlcA]$^+$<br>471.3464[M+H-2GlcA]$^+$<br>453.3368[M+H-2GlcA-H_2O]$^+$	UralsaponinB
92	9.930	$C_{41}H_{62}O_{14}$	777.4058(-0.4)	627.3535[M-H-Arab-H_2O]$^-$<br>449.1499[M-H-Arab-GlcA-H_2O]$^-$		647.3787[M+H-Arab]$^+$<br>453.3366[M+H-Arab-GlcA-H_2O]$^+$	Araboglycyrrhizin
93	10.021	$C_{20}H_{16}O_6$	351.0879(2.8)	283.0987[M-H-PA]$^-$	353.1031(1.7)	335.0930[M+H-H_2O]$^+$<br>311.0569[M+H-PP]$^+$	LicoisoflavoneB/Semilicoisoflavone B/allolicoisoflavone B
94	10.247	$C_{20}H_{18}O_6$	353.1024(-0.3)	297.0398[M-H-BT]$^-$	355.1187(1.4)	299.0583[M+H-BT]$^+$	Licoflavonol or its isomer
95	10.324	$C_{20}H_{20}O_6$	355.1185(0.8)	337.1077[M-H-H_2O]$^-$<br>293.0453[M-H-CO-2OH]$^-$<br>281.0457[M-H-H_2O-BT]$^-$<br>229.0868[M-H-BE]$^-$<br>125.0241[BE-H]$^-$		339.1212[M+H-H_2O]$^+$<br>283.0604[M+H-H_2O-BT]$^+$<br>301.0708[M+H-BT]$^+$	Uralenin
96	10.410	$C_{21}H_{20}O_6$	367.1173(-2.5)	339.1223[M-H-CO]$^-$<br>333.0754[M-H-2OH]$^-$	369.1342(1.1)	335.0929[M+H-2OH]$^+$<br>313.0712[M+H-BT]$^+$	Glycyrrhisoflavanone

				309.0397[M-H-CO-2CH$_3$]$^-$<br>297.0398[M-H-PI]$^-$			
97	10.483	$C_{21}H_{20}O_6$	367.1192(2.7)	337.1081[M-H-CH$_2$O]$^-$,<br>309.0408[M-H-CH$_2$O-CO]$^-$	369.1342(1.1)	313.0718[M+H-IB]$^+$,<br>285.0764[M+H-BT-CO]$^+$,<br>269.0478[M+H-BT-CO-CH$_4$]$^+$	Glycycoumarin
98	10.623	$C_{21}H_{24}O_5$	355.1549(1.1)	321.1107[M-H-2OH]$^-$<br>323.1283[M-H-CH$_4$O]$^-$<br>337.1086[M-H-H$_2$O]$^-$	357.1712(2.8)	301.1091[M+H-BT]$^+$;<br>321.1140[M+H-2H$_2$O]$^+$<br>313.1096[M+H-CO$_2$]$^+$	Glyasperins C
99	10.777	$C_{20}H_{18}O_6$	353.1022(-0.8)	297.0396[M-H-BT]$^-$<br>269.0458[M-H-BT-CO]$^-$	355.1190(2.3)	299.0564[M+H-BT]$^+$,<br>287.0574[M+H-IP]$^+$	Licoflavonol or its isomer
100	11.022	$C_{20}H_{18}O_6$	353.1020(-1.4)	309.1120[M-H-CO$_2$]$^-$<br>285.1125[M-H-IP]$^-$	355.1194(3.4)	340.0794[M+H-CH$_3$]$^+$<br>299.0556[M+H-BT]$^+$	Licoflavonol or its isomer
101	11.090	$C_{20}H_{18}O_6$	353.1025(0.0)	307.0964[M-H-CO-H$_2$O]$^-$<br>227.0707[M-H-BE]$^-$<br>125.0239[BE-H]$^-$	355.1183(0.3)	337.1064[M+H-H$_2$O]$^+$<br>179.0342[M+H-DCO]$^+$	Licoisoflavanone
102	11.165	$C_{20}H_{18}O_6$	353.1027(0.6)	325.1858[M-H-CO]$^-$	355.1190(2.3)	299.0558[M+H-BT]$^+$<br>327.1252[M+H-CO]$^+$	Licoflavonol or its isomer
103	11.248	$C_{21}H_{22}O_4$	337.1447(2.1)	281.0833[M-H-2CH$_3$-C$_2$H$_2$]$^-$<br>187.0768[M-H-2CH$_3$-OCD]$^-$	339.1600(1.2)	271.0978[M+H-IP]$^+$<br>245.1198[M+H-PH]$^+$<br>121.0299[M+H-MMEV]$^+$	Licochalcone A
104	11.353	$C_{21}H_{18}O_6$	365.1023(-0.5)	337.1076[M-H-CO]$^-$<br>307.0236[M-H-CO-2CH$_3$]$^-$	367.1172(-2.7)	339.1227[M+H-CO]$^+$<br>323.0545[M+H-CO$_2$]$^+$<br>311.0550[M+H-2CO]$^+$<br>283.0602[M+H-3CO]$^+$	Isoglycyrol

105	11.370	$C_{25}H_{26}O_7$	437.1595(-1.1)	381.1332[M-H-BT]$^-$<br>337.1071[M-H-MPEO]$^-$<br>307.0240[M-H-MPEO-2CH$_3$]$^-$	439.1745(-2.7)	383.1125[M+H-BT]$^+$	Kanzonol T
106	11.816	$C_{20}H_{16}O_5$	335.0922(0.9)	320.0675[M-H-CH$_3$]$^-$<br>305.0450[M-H-2CH$_3$]$^-$<br>291.1021[M-H-CHO-CH$_3$]$^-$	337.1076(0.0)	319.0964[M+H-H$_2$O]$^+$<br>295.0607[M+H-PP]$^+$	Glabrone
107	11.850	$C_{12}H_{14}O_2$			191.1074(1.0)	173.0964[M+H-H$_2$O]$^+$<br>145.1011[M+H-HCOOH]$^+$<br>117.0699[M+H-HCOOH-C$_2$H$_4$]$^+$	Z-/E-ligustilide
108	12.016	$C_{20}H_{18}O_6$	353.1023(-0.6)	323.1281[M-H-2CH$_3$]$^-$<br>269.0445[M-H-2DO]$^-$<br>241.0493[M-H-2DO-CO]$^-$	355.1195(3.7)	299.0556[M+H-BT]$^+$	Glyasperin F
109	12.314	$C_{20}H_{16}O_6$	351.0872(0.9)	336.0628[M-H-CH$_3$]$^-$<br>307.0968[M-H-CO$_2$]$^-$<br>283.0972[M-H-PA]$^-$<br>241.0862[M-H-RL]$^-$	353.1026(0.3)	335.0917[M+H-H$_2$O]$^+$<br>311.0558[M+H-PP]$^+$<br>299.0558[M+H-3H$_2$O]$^+$<br>283.0614[M+H-PI]$^+$	Demethylglycyrol
110	12.765	$C_{20}H_{16}O_5$	335.0915(-1.2)	317.0812[M-H-H$_2$O]$^-$<br>305.0452[M-H-2CH$_3$]$^-$<br>291.0655[M-H-CO$_2$]$^-$	337.1074(-0.6)	295.0581[M+H-PP]$^+$	Isoglabrone
111	12.879	$C_{25}H_{28}O_4$	391.1915(1.5)	203.0716[M-H-MBV]$^-$<br>187.1129[MBV-H]$^-$<br>132.0577[MBV-BT-H]$^-$	393.2063(-0.8)	337.1436[M+H-BT]$^+$	Glabrol
112	13.280	$C_{22}H_{26}O_5$	369.1704(0.5)	339.1235[M-H-CH$_2$O]$^-$<br>311.0923[M-H-CH$_2$O-CO]$^-$	371.1841(-4.6)	315.1224[M+H-BT]$^+$<br>235.1329[M+H-MLT]$^+$	Glyasperins D

				279.0654[M-H-CH_2O-CO-CH_3OH]$^-$<br>135.0449[M-H-DOT]$^-$			
113	13.394	$C_{25}H_{28}O_6$	423.1821(3.1)	367.1166[M-H-BT]$^-$<br>229.0867[M-H-BF]$^-$<br>193.0867[BF-H]$^-$		369.1359[M+H-BT]$^+$<br>365.1031[M+H-ISP]$^+$<br>351.1235[M+H-ISD]$^+$<br>221.1181[M+H-APP]$^+$<br>191.1073[M+H-APP-2CH_3]$^+$	Glyinflanin F
114	13.577	$C_{25}H_{28}O_5$	407.1862(1.0)	337.1442[M-H-PI]$^-$<br>219.0658[M-H-HMDB]$^-$<br>187.1122[HMDB-H]$^-$	409.2012(-0.7)	353.1389[M+H-BT]$^+$<br>191.1072[HMBB+H]$^+$	Glycyrdione A
115	13.708	$C_{25}H_{26}O_6$	421.1647(-0.9)	352.0945[M-H-C_5H_9]$^-$<br>309.0397[M-H-4CO]$^-$	423.1805(-0.7)	367.1177[M+H-BT]$^+$<br>311.0555[M+H-2BT]$^+$<br>261.1852[M+H-DCE]$^+$<br>163.0756[DCE+H]$^+$	6,8-diprenylorobol
116	13.743	$C_{24}H_{28}O_4$			381.2065(-0.3)	335.2004[M+H-H_2O-CO]$^+$<br>191.1071[M+H]-ZBD]$^+$<br>173.0963[M+H-ZBD-H_2O]$^+$<br>145.1013[M+H-ZBD-HCOOH]$^+$	Senkyunolide O or its isomer
117	13.908	$C_{25}H_{28}O_6$	423.1799(-2.1)	299.0861[M-H-MBT]$^-$<br>193.0862[MBT-H]$^-$	425.1959(-1.2)	369.1337[M+H-BT]$^+$<br>191.1073[M+H-DMP]$^+$<br>135.0443[M+H-DMP-BT]$^+$	GancaoninE/ 3’-(γ,γ-dimethylally1)-kievitone
118	14.040	$C_{25}H_{26}O_6$	421.1646(-1.2)	309.0398[M-H-2BT]$^-$	423.1812(0.9)	367.1180[M+H-BT]$^+$	Glyurallin B/Isoangustone A /Angustone A

						311.0553[M+H-2BT]$^+$<br>283.0581[M+H-2BT-CO]$^+$<br>255.0659[M+H-2BT-2CO]$^+$	
119	14.537	$C_{30}H_{46}O_4$	469.3306(-2.6)	425.3416[M-H-CO_2]$^-$<br>355.2609[M-H-CO_2-ME]$^-$	471.3479(1.1)	453.3398[M+H-H_2O]$^+$<br>425.2890[M+H-HCOOH]$^+$<br>407.2775[M+H-HCOOH-H_2O]$^+$	Glycyrrhetic acid
120	14.606	$C_{25}H_{26}O_6$	421.1646(-1.2)	193.0862[MBT-H]$^-$			Glyurallin B/Isoangustone A /Angustone A
121	14.634	$C_{24}H_{28}O_4$			381.2062(-1.0)	363.1953[M+H-H_2O]$^+$<br>335.2003[M+H-H_2O-CO]$^+$<br>191.1071[M+H-ZBD]$^+$<br>173.0966[M+H-ZBD-H_2O]$^+$	Tokinolide B or its isomer
122	14.852	$C_{24}H_{28}O_4$			381.2070(1.0)	363.1960[M+H-H_2O]$^+$<br>335.2015[M+H-H_2O-CO]$^+$<br>191.1076[M+H-ZBD]$^+$<br>173.0966[M+H-ZBD-H_2O]$^+$	Levistolide A
123	14.966	$C_{24}H_{28}O_4$			381.2063(-0.8)	363.1958[M+H-H_2O]$^+$<br>335.2008[M+H-H_2O-CO]$^+$<br>191.1082[M+H-ZBD]$^+$<br>173.0966[M+H-ZBD-H_2O]$^+$	Angelicide or its isomer
124	15.052	$C_{24}H_{28}O_4$			381.2068(0.5)	363.1956[M+H-H_2O]$^+$<br>191.1077[M+H-ZBD]$^+$<br>173.0965[M+H-ZBD-H_2O]$^+$	Z,Z′-6.8′,7.3′-Diligustilide or its isomer
125	15.481	$C_{24}H_{28}O_4$			381.2057(-2.4)	191.1071[M+H-ZBD]$^+$	Z-Ligustilide dimer E-232 or its

							isomer
						173.0959[M+H-ZBD-H_2O]$^+$	
126	15.601	$C_{39}H_{54}O_6$	617.3837(-0.8)	573.3922[M-H-CO_2]$^-$<br>453.3256[M-H-HDPA]$^-$	619.3985(-2.3)	601.3856[M+H-H_2O]$^+$<br>455.3493[M+H-HDPA]$^+$<br>437.3414[M+H-HDPA-H_2O]$^+$	p-Coumaroyl maslinic acid
127	15.681	$C_{24}H_{28}O_4$			381.2047(-5.0)	363.1944[M+H-H_2O]$^+$<br>191.1069[M+H-ZBD]$^+$<br>173.0961[M+H-ZBD-H_2O]$^+$<br>145.1018[M+H-ZBD-HCOOH]$^+$	Neodiligustilide or its isomer
128	15.932	$C_{25}H_{24}O_6$	419.1495(0.0)	404.1255[M-H-CH_3]$^-$<br>375.1601[M-H-CO_2]$^-$<br>219.0655[M-H-EDC]$^-$<br>199.0754[EDC-H]$^-$		403.1885[M+H-H_2O]$^+$<br>365.1022[M+H-BT]$^+$<br>347.0907[M+H-BT-H_2O]$^+$	gancaonin H
129	16.098	$C_{24}H_{28}O_4$			381.2064(-0.5)	363.1938[M+H-H_2O]$^+$<br>335.2002[M+H-H_2O-CO]$^+$<br>191.1073[M+H-ZBD]$^+$<br>173.0967[M+H-ZBD-H_2O]$^+$	Z,Z′-3.3′,8.8′-Diligustilide or its isomer
130	16.195	$C_{24}H_{28}O_4$			381.2068(0.5)	191.1067[M+H-ZBD]$^+$<br>173.0962[M+H-ZBD-H_2O]$^+$<br>145.1012[M+H-ZBD-HCOOH]$^+$	E,E′-3.3′, 8.8′-Diligustilide or its isomer
131	16.292	$C_{24}H_{28}O_4$			381.2062(-1.0)	363.1955[M+H-H_2O]$^+$	E,E′-3.8′, 8·3′-Isodiligustilide or its isomer

				335.2032[M+H-H_2O-CO]$^+$<br>191.1073[M+H-ZBD]$^+$<br>173.0963[M+H-ZBD-H_2O]$^+$	
132	17.390	$C_{24}H_{28}O_4$	381.2059(-1.8)	363.1923[M+H-H_2O]$^+$<br>335.2007[M+H-H_2O-CO]$^+$<br>191.1071[M+H-ZBD]$^+$<br>173.0965[M+H-ZBD-H_2O]$^+$	3,3′Z-6.7′,7.6'-Diligustilide or its isomer
133	17.521	$C_{24}H_{28}O_4$	381.2062(-1.0)	363.1971[M+H-H_2O]$^+$<br>335.1998[M+H-H_2O-CO]$^+$<br>191.1070[M+H-ZBD]$^+$<br>173.0964[M+H-ZBD-H_2O]$^+$	3α.7′α,7α.3′α-Diligustilide or its isomer
134	17.784	$C_{24}H_{28}O_4$	381.2063(-0.8)	363.1975[M+H-H_2O]$^+$<br>335.2022[M+H-H_2O-CO]$^+$<br>191.1069[M+H-ZBD]$^+$<br>173.0958[M+H-ZBD-H_2O]$^+$	(3′)-(3R, 8S, 3′aR, 6′S)-3.3a′, 8.6′-Biligustilide or its isomer

Glc: Glucose, ADE: Adenine, HDTDO: (1aS,1bS,3S,5aR,6aS)-3-hydroxytetrahydro-5H-oxireno[2',3':4,5]furo[3,2-d][1,3,2]dioxaphosphinine 3-oxide, DTFD: (4aR,7S,7aS)-2,7-dihydroxytetrahydro-4H-furo[3,2-d][1,3,2]dioxaphosphinin-6-ide 2-oxide, GA: Gallic acid, BZ: Benzoic Acid, 4-OH-BZ: 4-hydroxyl-benzoic acid , 2-OH-BZ: 2-hydroxyl-benzoic acid , HB: 4-hydroxy-3-methoxybenzoic acid, VP: 4-vinylphenol, Api:apiose, MB: methyl benzoate , MG: ((2R,3S,4R,5S)-3,4,5-trihydroxytetrahydro-6l2-pyran-2-yl)methyl 3,4,5-trihydroxybenzoate, Arab: Arabinose, BH:2-o-Benzaldehyde, RL:resorcinol, HM: 3-hydroxy-3-methylglutaroyl, Rha: rhamnose, PC: P-coumaroyl, GlcA: glucuronic acid, PM:2-methoxy-coumaroyl, PE: 3-methoxy-5-(3l2-propyl)benzene-1,2-diol, BT: isobutylene, MVD: 3-methoxy-4-vinylbenzene-1,2-diol, PP: propylene, PA: 3-methylbuta-1,2-diene, BE: benzene-1,3,5-triol, PI: pentanol-iosprene, IP: isoprene, DCO: 2,2-dimethyl-2H-chromen-5-ol, OCD: 4-oxomethylidenecyclohexa-2,5-dienone, PH: phenol, MMEV: 5-methoxy-2-(2-methylbut-3-en-2-yl)-4-vinylphenol, MPEO:

2-methylpent-4-en-2-ol, PP: propylene, 2DO:2,2-dimethyl-2H-oxete, MBV: 2-(3-methylbut-2-en-1-yl)-4-vinylphenol, DOT: 2,4-dimethoxy-3-(3-methylbut-2-en1yl)-7-oxabicyclo[4.2.0]octa-1,3,5-triene, MLT: 4-vinylbenzene-1,3-diol, BF: 2-(2-hydroxypropan-2-yl)-2,3-dihydrobenzofuran-6-ol, APP: 1-(4-hydroxy-3-(3-methylbut-2-en-1-yl)phenyl)ethan-1-one, ISD: 2-methylpropan-2-ol, ISP: propan-2-ol, HMDB:4-hydroxy-3-(3-methylbuta-1,2-dien-1-yl)benzaldehyde, HMBB:4-hydroxy-3-(3-methylbut-2-en-1-yl)benzaldehyde, DCE: 2-(3,4-dihydroxyphenyl)cycloprop-2-en-1-one, ZBD: (Z)-3-butylidene-4,5-dihydroisobenzofuran-1(3H)-one, DMP: 2-(2,4-dihydroxy-3-(3-methylbut-2-en-1-yl)phenyl)propanal, MBT: 2-(3-methylbut-2-en-1-yl)benzene-1,3,5-triol, ME:3-methylbut-1-ene, HDPA: (E)-3-(4-hydroxyphenyl)acrylic acid, EDC: 6-ethynyl-2,2-dimethyl-2H-chromen-8-ol

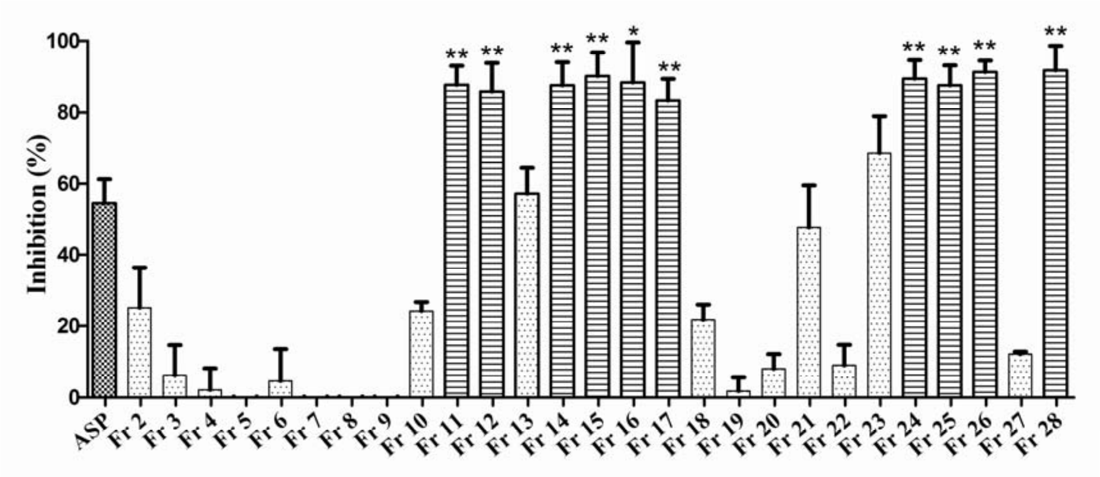

Supplementary Figure S1. The anti-platelet effects of the fractions from DJF. Significant difference with respect to aspirin (ASP): *, $p < 0.05$; **, $p < 0.01$.

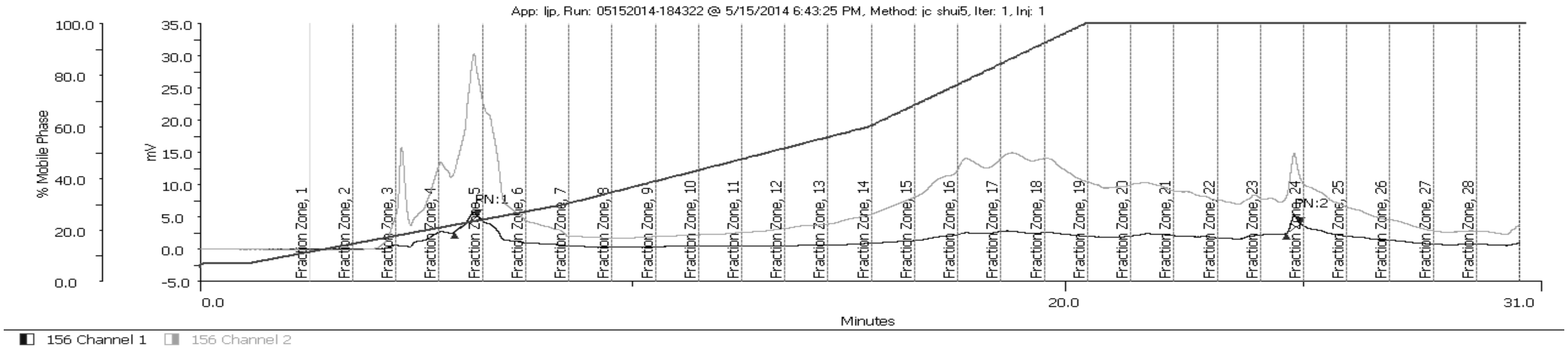

Supplementary Figure S2. The preparative chromatogram of DJF extractions.

Supplementary Table S2. The potential target proteins in the DT network.

Protein name	Uniprot ID
Thrombin	P00734
Thromboxane A2 receptor	P21731
Proteinase-activated receptor 1	P25116
Proteinase-activated receptor 4	Q96RI0
P2Y purinoceptor 1	P47900
P2Y purinoceptor 12	Q9H244
Prostacyclin receptor	P43119
Cytosolic phospholipase A2	P47712
Guanine nucleotide-binding protein G(q) subunit a	P50148
Guanine nucleotide-binding protein G(s) subunit a	P63092
Phosphatidylinositol 4,5-bisphosphate 3-kinase catalytic subunit a	P42336
Phosphatidylinositol 4,5-bisphosphate 3-kinase catalytic subunit b	P42338
Phosphatidylinositol 4,5-bisphosphate 3-kinase catalytic subunit g	P48736
Phosphatidylinositol 4,5-bisphosphate 3-kinase catalytic subunit d	O00329
Phosphatidylinositol 3-kinase regulatory subunit a	P27986
Phosphatidylinositol 3-kinase regulatory subunit b	O00459
Phosphatidylinositol 3-kinase regulatory subunit g	Q92569
Phosphatidylinositol 3-kinase regulatory subunit 5	Q8WYR1
1-phosphatidylinositol 4,5-bisphosphate phosphodiesterase b-1	Q9NQ66
1-phosphatidylinositol 4,5-bisphosphate phosphodiesterase b-2	Q00722
1-phosphatidylinositol 4,5-bisphosphate phosphodiesterase b-3	Q01970
1-phosphatidylinositol 4,5-bisphosphate phosphodiesterase b-4	Q15147
Adenylate cyclase type 1	Q08828
Adenylate cyclase type 2	Q08462
Adenylate cyclase type 3	O60266
Adenylate cyclase type 4	Q8NFM4
Adenylate cyclase type 5	O95622
Adenylate cyclase type 6	O43306
Adenylate cyclase type 7	P51828
Adenylate cyclase type 8	P40145
Adenylate cyclase type 9	O60503
Lymphocyte cytosolic protein 2	Q13094
1-phosphatidylinositol 4,5-bisphosphate phosphodiesterase g-2	P16885
Tyrosine-protein kinase BTK	Q06187
Prostaglandin G/H synthase 1 (COX-1)	P23219
Protein kinase C	P41743
RAS guanyl-releasing protein 1	O95267
RAS guanyl-releasing protein 2	Q7LDG7
cAMP-dependent protein kinase catalytic subunit a	P17612
Thromboxane-A synthase	P24557
Ras-related protein Rap-1A	P62834
Ras-related protein Rap-1b	P61224
Vasodilator-stimulated phosphoprotein	P50552
Amyloid b A4 precursor protein-binding family B member 1-interacting protein	Q7Z5R6
Actin, cytoplasmic 1	P60709
Fermitin family homolog 3	Q86UX7
Talin-1	Q9Y490
Talin-2	Q9Y4G6
Integrin a-IIb	P08514
Integrin b-3	P05106
Fibrinogen a chain	P02671
Fibrinogen b chain	P02675

Fibrinogen g chain	P02679
Guanine nucleotide-binding protein G(i) subunit a-1	P63096
Synaptosomal-associated protein 23	O00161
Vesicle-associated membrane protein 8	Q9BV40

Supplementary Table S3. The elution condition for DJF fractionating

Time (min)	A%	B%
0	95	5
8	70	30
15	40	60
20	0	100
30	0	100

Supplementary Table S4. The elution condition for ingredient identification

Time(min)	A%	B%
0	95	5
20	5	95
22	5	95

Supplementary Table S5. The elution condition for the plasma concentration testing

Time (min)	A%	B%
0	95	5
3	85	15
8	75	25
36	5	95
37	0	100
40	0	100

Supplementary Table S6. The composition of the Tris buffer solution.

Chemicals	Concentration (mM)	Weight (g)
NaCl	130	3.042
Sodium citrate	10	1.1764
$NaHCO_3$	9	0.3024
Glucose	6	0.432
$MgCl_2$	0.9	0.0342
KH_2PO_4	0.81	0.04406
Tris-base	10	0.4844

Supplementary Table S7. The composition of the ACD solution.

Chemicals	Concentration (mM)	Weight (g)
Sodium citrate	75	0.882
Glucose	124	0.893
Citrate acid	38	0.292

Supplementary Table S8. The grouping of the platelet aggregation experiments.

Group	Constituent
Negative	100mL platelets + 50mL buffer solution + 50mL (agonist and CaCl2)
Positive	100mL platelets + 50mL reagent solution + 50mL (agonist and CaCl2)
Investigating	100mL platelets + 50mL compound solution + 50mL (agonist and CaCl2)
Control	150mL buffer solution + 50mL compound solution

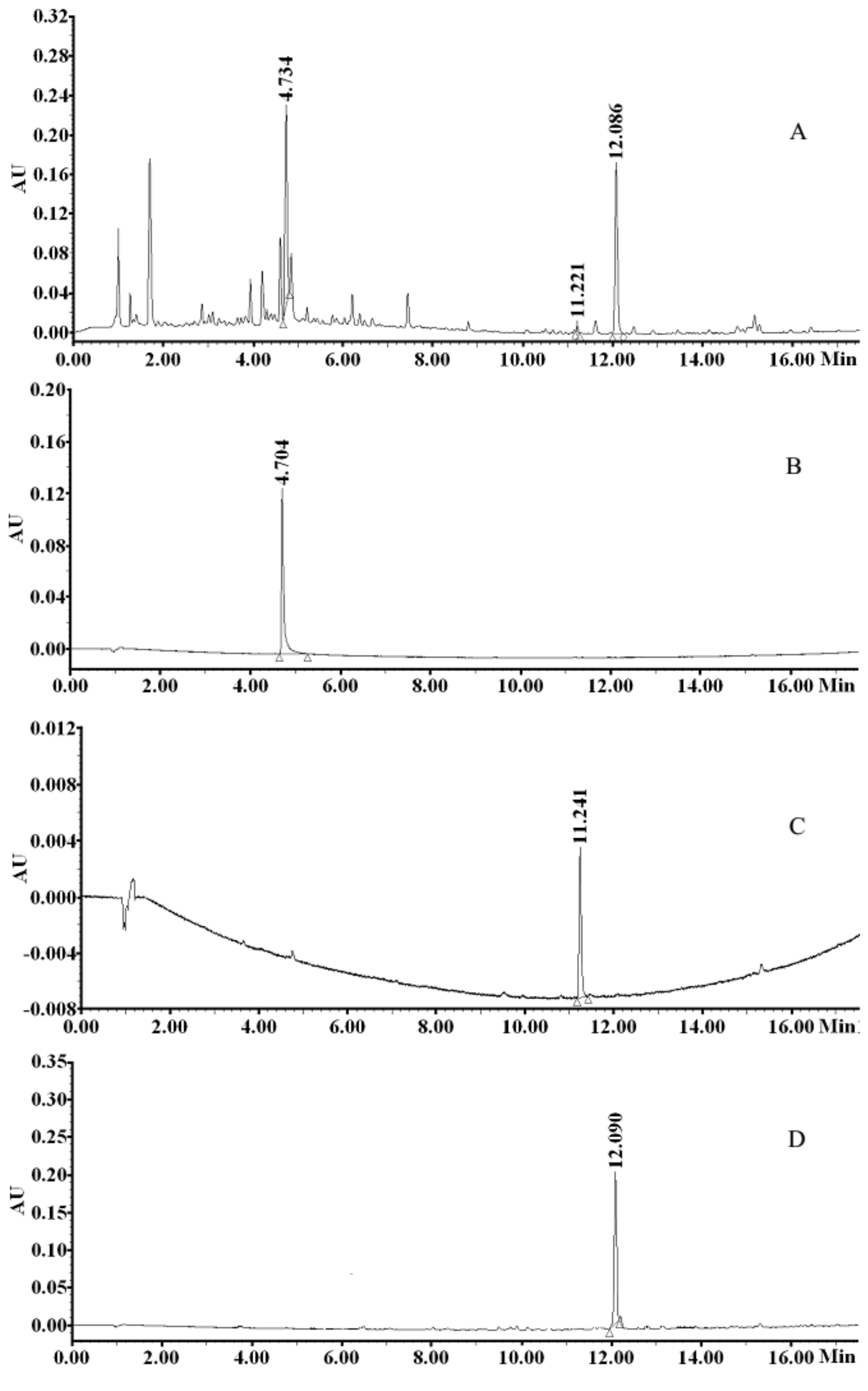

Supplementary Figure S3. The UPLC-DAD chromatograms of DJF, showing the full formula (A, 40.88mg/mL), along with the data of the standard solutions of Pentagalloylglucose (B, 28.9μg/mL), Licochalcone A (C, 0.96μg/mL) and (Z)-ligustilide (D, 128.0μg/mL).

Supplementary Table S9. The composition of the 3-compound formulae

Composition / Groups	Pentagalloylglucose	Licochalcone A	(*Z*)-Ligustilide
RF_1-C_1	46.25μg/mL	46.25μg/mL	47.50μg/mL
RF_1-C_2	23.13μg/mL	23.13μg/mL	23.75μg/mL
RF_1-C_3	11.57μg/mL	11.57μg/mL	11.88μg/mL
RF_2-C_1	45.00μg/mL	1.25μg/mL	155.00μg/mL
RF_2-C_2	22.50μg/mL	0.63μg/mL	77.50μg/mL
RF_2-C_3	11.25μg/mL	0.32μg/mL	38.75μg/mL
